# An *Irf6*-*Esrp1/2* regulatory axis controls midface morphogenesis in vertebrates

**DOI:** 10.1242/dev.194498

**Published:** 2020-12-23

**Authors:** Shannon H. Carroll, Claudio Macias Trevino, Edward B. Li, Kenta Kawasaki, Nikita Myers, Shawn A. Hallett, Nora Alhazmi, Justin Cotney, Russ P. Carstens, Eric C. Liao

**Affiliations:** 1 Center for Regenerative Medicine, Massachusetts General Hospital, Boston, MA 02114, USA; 2 Shriners Hospital for Children, Boston, MA 02114, USA; 3 Harvard Medical School, Boston, MA 02115, USA; 4 Harvard School of Dental Medicine, Boston, MA 02115, USA; 5 Division of Plastic and Reconstructive Surgery, Massachusetts General Hospital, Boston, MA 02114, USA; 6 Department of Medicine, Perelman School of Medicine, University of Pennsylvania, Philadelphia, PA 19104, USA; 7 Department of Genetics and Genome Sciences, University of Connecticut Health, CT 06030, USA

**Keywords:** *IRF6*, *ESRP1*, Craniofacial, Cleft, Development

## Abstract

*Irf6* and *Esrp1* are important for palate development across vertebrates. In zebrafish, we found that *irf6* regulates the expression of *esrp1*. We detailed overlapping *Irf6* and *Esrp1/2* expression in mouse orofacial epithelium. In zebrafish, *irf6* and *esrp1/2* share expression in periderm, frontonasal ectoderm and oral epithelium. Genetic disruption of *irf6* and *esrp1/2* in zebrafish resulted in cleft of the anterior neurocranium. The *esrp1/2* mutant also developed cleft of the mouth opening. Lineage tracing of cranial neural crest cells revealed that the cleft resulted not from migration defect, but from impaired chondrogenesis. Analysis of aberrant cells within the cleft revealed expression of *sox10*, *col1a1* and *irf6*, and these cells were adjacent to *krt4*^+^ and *krt5*^+^ cells. Breeding of mouse *Irf6*; *Esrp1*; *Esrp2* compound mutants suggested genetic interaction, as the triple homozygote and the *Irf6*; *Esrp1* double homozygote were not observed. Further, *Irf6* heterozygosity reduced *Esrp1/2* cleft severity. These studies highlight the complementary analysis of *Irf6* and *Esrp1/2* in mouse and zebrafish, and identify a unique aberrant cell population in zebrafish expressing *sox10*, *col1a1* and *irf6*. Future work characterizing this cell population will yield additional insight into cleft pathogenesis.

## Abstract (precis)

**Summary:** Experiments using complementary models indicate that disruption of *Irf6* and *Esrp1/2* in mouse and zebrafish results in orofacial clefts, with an aberrant mesenchymal/epithelial cell population identified in the cleft of the *esrp1/2* zebrafish mutant.

## INTRODUCTION

Development of vertebrate craniofacial structures requires coordinated cellular induction, migration, proliferation and differentiation, which allow for the positioning of adjacent epithelial-lined facial processes that ultimately merge (Reid et al., 2011; O'Donoghue et al., 2020; Knight and Schilling, 2006; Jiang et al., 2006; Helms et al., 2005; Dougherty et al., 2012; Creuzet et al., 2005; Cordero et al., 2011; Abramyan and Richman, 2015). Morphogenesis of facial structures such as the midface, lip and palate requires convergence of the medial and lateral nasal prominences and the fusion of the secondary palatal shelves at the midline (Losa et al., 2018; Jiang et al., 2006; Abramyan and Richman, 2015). Failure of these processes to fuse results in orofacial clefts of the lip, primary palate or secondary palate (Gritli-Linde, 2008). Orofacial clefts are among the most common congenital structural anomalies (Yuan et al., 2011; Juriloff and Harris, 2008; Goodwin et al., 2015). From genome-wide association studies carried out over a decade ago to more recent whole-genome sequencing projects of orofacial cleft cohorts, cleft-associated genetic loci continue to be identified, and the transcription factor *IRF6* is one of the most commonly associated genes (Zucchero et al., 2004; Marazita, 2012; Yu et al., 2017; Cox et al., 2018). *IRF6* disruption is causal for syndromic cleft in Van der Woude and popliteal pterygium syndromes, and associated with non-syndromic orofacial clefts (Zucchero et al., 2004; Leslie et al., 2013; Kondo et al., 2002; Beaty et al., 2016).

Several IRF6 transcriptional targets – such as *GRHL3*, *WDR65* (*CFAP57*), *OVOL1* and *KLF4* – have been identified, which are also important for palate development and implicated in human cleft pathogenesis (Rorick et al., 2011; Liu et al., 2016; Kousa and Schutte, 2016; de la Garza et al., 2013). These studies support the premise that investigation of *Irf6* and its transcriptional network will identify key genes that regulate palate development. Multiple mouse models have been generated to investigate *Irf6* function, including a total *Irf6* knockout and substitution of key functional residue *Irf6*^R84C^ in the DNA-binding domain (Richardson et al., 2006; Ingraham et al., 2006). These *Irf6* mutant mouse models exhibited disrupted epithelial terminal differentiation and lack of a functional periderm, leading to pathological adhesions of epithelial embryonic tissues (Richardson et al., 2006; Iwata et al., 2013; Ingraham et al., 2006; Ferretti et al., 2011). The epithelial differentiation and adhesion defects are thought to prevent elevation of the palatal shelves, and ultimately these mice develop a cleft in the secondary palate. Additionally, the midface of these mice were hypoplastic, a phenotype that was attributed to the dysfunctional embryonic epithelium (Richardson et al., 2006; Ingraham et al., 2006).

Epithelial splicing regulatory proteins 1 and 2 (*Esrp1*, *Esrp2*) are also important in embryonic epithelial differentiation and palate development (Lee et al., 2020, 2018; Bebee et al., 2015). *Esrp2* and its homolog *Esrp1* are regulators of RNA splicing that are specifically expressed in the epithelium (Warzecha et al., 2009). *Esrp1/2* knockout mice exhibit bilateral cleft of the lip and primary palate, as well as a secondary palate cleft (Bebee et al., 2015). *Esrp1/2* are unusual among regulators of RNA splicing in that they are tissue restricted and exhibit dynamic expression during embryogenesis (Burguera et al., 2017; Bebee et al., 2015). The developmental importance of *Esrp1/2* is underscored by their conservation across species, from ascidians to zebrafish, *Xenopus*, mouse and humans (Burguera et al., 2017). Gene variant in *ESRP2* was also recently reported in human orofacial cleft cohorts (Cox et al., 2018).

The mouse has been an important experimental model to study craniofacial and palate morphogenesis (Gritli-Linde, 2008). Secondary palate development in the mouse is similar to that in humans, with the analogous stages of vertical outgrowth, elevation, horizontal growth and fusion (Juriloff and Harris, 2008; Gritli-Linde, 2008). Many genes associated with cleft lip and palate (CL/P) in humans, when disrupted in the mouse, result in cleft of the secondary palate without affecting morphogenesis of the primary palate and lip (Van Otterloo et al., 2016; Gritli-Linde, 2008). So while the mouse model can be useful to study the secondary palate, the use of mouse models to study cleft of the lip and primary palate has been less effective as there are remarkably few mouse models in which development of the lip and primary palate are perturbed (Gritli-Linde, 2008). Meanwhile, clinically, CL/P is more common than isolated cleft of the palate only (CPO), and human genetic studies have suggested that the genetics underpinning CL/P and CPO are distinct (Juriloff and Harris, 2008; Gritli-Linde, 2008). The developmental processes of outgrowth of the facial prominences followed by convergence and fusion are thought to be conserved across mammals (Abramyan and Richman, 2015). Therefore, it is hypothesized that differences in mouse versus human phenotypic presentation are caused by spatiotemporal differences in craniofacial development (Gritli-Linde, 2008). In this context, the phenotype of bilateral clefts affecting the lip, primary and secondary palate in the *Esrp1/2* mutant mouse is unique among mouse models and is a valuable tool to study lip and palate morphogenesis.

Zebrafish has been favored by embryologists as an animal model to study craniofacial development, owing to its accessibility, transparency and genetic tractability (Schilling and Le Pabic, 2009; Lieschke and Currie, 2007; Kimmel, 1989). Although a secondary palate, which partially or entirely separates the oral and nasal cavities, is reserved to amniotes, the primary palate is appreciably conserved across vertebrates (Abramyan and Richman, 2015). The primary palate establishes the intact upper jaw (Abramyan and Richman, 2015), which in the larval zebrafish consists of the ethmoid plate, also known as the anterior neurocranium (ANC). In all vertebrates, the most anterior cranial neural crest cells (CNCCs), that migrate rostral then turn caudal and ventral to the eye, contribute to the median frontonasal prominence, and a second CNCC stream, that migrates inferior to the eye and into the first pharyngeal arch, generates the paired maxillary prominences (Reid et al., 1986; Swartz et al., 2011; Kimmel, 1989; Dougherty et al., 2012). The ANC of the zebrafish is formed from the convergence of the median element, which is derived from the frontonasal prominence, and paired lateral elements that are derived from the maxillary prominences (Swartz et al., 2011; O'Donoghue et al., 2020; Duncan et al., 2017). Zebrafish homologs of human genes associated with orofacial clefts will disrupt morphology of the ANC, as have been observed for a number of genes, such as *capzb*, *pitx2*, *pdgfra*, *smad5*, *tgfb2*, *fgf10a* and *wnt9a* (O'Donoghue et al., 2020; Van Otterloo et al., 2016; Duncan et al., 2017).

Here, we carried out detailed gene expression analysis of *Irf6*, *Esrp1* and *Esrp2* in mouse and zebrafish in order to understand the comparative morphogenesis of facial structures and periderm between these important vertebrate genetic models. We analyzed and compared the *Irf6* and *Esrp1/2* mutant phenotypes to elucidate the comparative morphologies and genetic epistasis between these genes. Further, we generated zebrafish *irf6* and *esrp1/2* zebrafish mutants and examined their requirement in morphology of the stomodeum opening and ANC. Interestingly, we identified an aberrant cell population with epithelial and mesenchymal molecular signatures that localized to the region of the ANC cleft. This work highlights the relative strengths of the mouse and zebrafish models for investigating the morphogenetic mechanisms of orofacial clefts, and contributes new insights into the function of *Irf6* and *Esrp1/2* during palatogenesis.

## RESULTS

### *irf6* null zebrafish embryos have decreased expression of *esrp1*

**Fig. 1.**
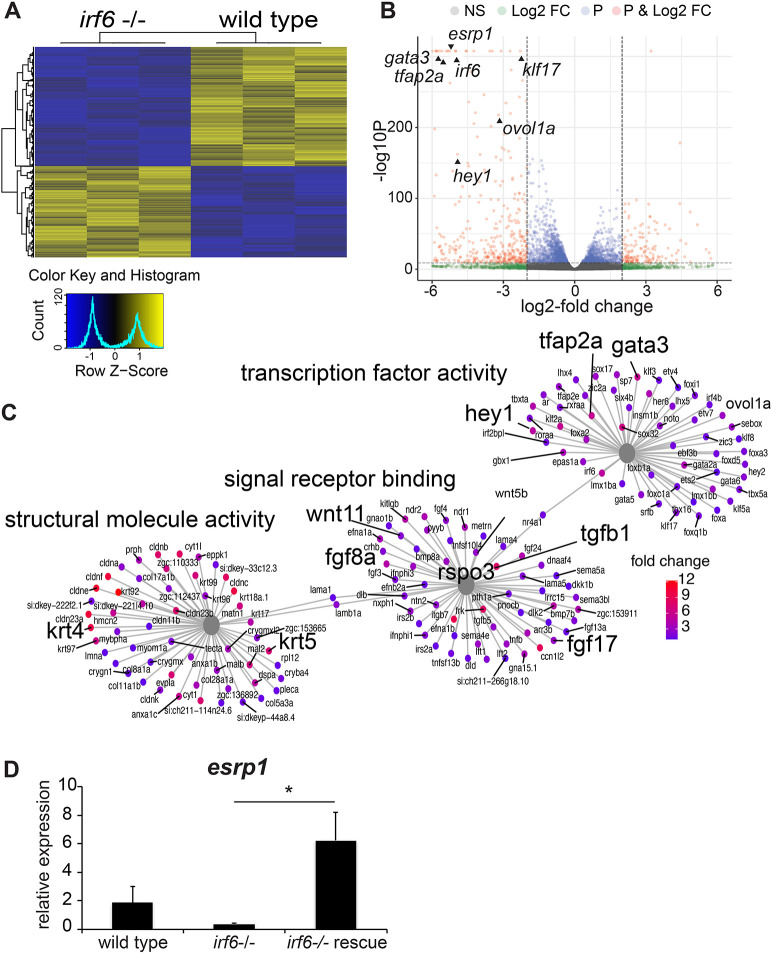
***esrp1* expression is downregulated in *irf6* null zebrafish embryos.** (A) Hierarchical clustering of top differentially expressed genes (DEGs) defined by RNA-seq performed on wild-type (WT) versus mz-*irf6*^-8bp/-8bp^ (*irf6^−/−^*) zebrafish embryos at 4-5 hpf. Top DEGs were identified by selecting genes with an adjusted *P*-value (Benjamini–Hochberg) <0.01 and absolute log2-fold change >2. Data are shown for three biological replicates. Color scale on the bottom left represents relative levels of expression, with yellow showing higher expression levels and blue showing lower expression. (B) Volcano plot from the RNA-seq dataset, showing the distribution of DEGs based on *P*-values (P) and log2-fold change (Log2 FC). NS, not significant. Previously published *irf6*-regulated genes are expressed at significantly higher levels in WT relative to mz-*irf6*^−/−^, including *grhl3*, *klf17* and *wnt11*. The newly identified cleft-associated gene *esrp1* is also expressed significantly higher in WT relative to *irf6*^−/−^. Vertical dashed lines represent the *P*-value cutoff of 0.01 and the log2-fold change cutoff of 2, respectively. (C) Gene ontology (GO) gene-concept network analysis of RNA-seq data, showing that *irf6*^−/−^ embryos have perturbations in processes such as transcription factor activity, signal receptor binding and structural molecule activity. Note that many of these genes – such as *wnt11*, *fgf8*, *tgfb1*, *krt4* and *krt5* – are implicated in ectoderm development and cell specification. Gray nodes show GO terms, colored nodes show individual genes from the RNA-seq dataset, and black lines connect genes to one or more associated GO terms. Colored nodes show relative enrichment (measured by fold change) of genes in WT samples relative to *irf6^−/−^* embryos. Maps were generated using the enrichplot package in R. (D) qPCR gene expression analysis for *esrp1*, showing ∼80% downregulation in mz-*irf6*^-8bp/-8bp^ embryos compared with WT at 4 hpf, and rescued *esrp1* gene overexpression in mz-*irf6*^-8bp/-8bp^ embryos injected with WT zebrafish *irf6* mRNA. *n*=4. Unpaired Student’s *t*-test, *P<0.05.

We previously generated a functionally null *irf6* zebrafish allele (Li et al., 2017). Using CRISPR/Cas9, an 8 bp deletion in exon 6 of the *irf6* coding region resulting in a frameshift and premature stop codon, leading to the ablation of *irf6* function. It was observed that embryos lacking maternally expressed *irf6* exhibited epiboly arrest and periderm rupture at 4-5 h post-fertilization (hpf) (Li et al., 2017). Utilizing this *irf6* null model, we aimed to identify genes that were differentially downregulated in *irf6* null versus wild-type (WT) embryos. We performed RNA sequencing (RNA-seq) on WT and maternal/zygotic *irf6* null (mz-*irf6*^-8bp/-8bp^) embryos at 4.5 hpf, just before embryo rupture at the onset of gastrulation. Differential expression analysis revealed a substantial number of significantly differentially expressed genes (DEGs; *n*=10,299, adjusted *P*-value <0.05) (Fig. 1A-C). Full differential expression results are available in Table S1. To visualize changes in this large number of DEGs, a heat map was generated, which illustrates 1377 upregulated and 1799 downregulated genes with an absolute fold change greater than 2 in *irf6* null relative to WT (Fig. 1A). The patterns of gene expression among these strongly DEGs were highly reproducible across the two genotypes, and demonstrated relatively similar numbers of downregulated and upregulated genes. When we visualized DEGs using significance values relative to fold change in expression, we found that the most significant and strongest effects on gene expression were biased toward those downregulated in the *irf6* null embryos (Fig. 1B). The RNA-seq results revealed significant downregulation of genes previously known to be downregulated with disruptions in *irf6* function (Fig. 1B,C). Disruption of *irf6* via injecting dominant-negative *irf6* mRNA led to downregulation of many periderm-enriched genes [including *grhl1*, *krt5*, *krt18* (*krt18a.1*), *tfap2a* and *klf2b*] and genes for adhesion molecules (including claudins and cadherins) (de la Garza et al., 2013). Here, we found a similar expression profile in the mz-*irf6*^-8bp/-8bp^ embryos relative to WT (Fig. 1B,C).

To further understand the molecular pathways and biological functions being affected in the *irf6* null embryo we performed gene ontology (GO) analyses on upregulated and downregulated gene sets. Of particular interest were significant changes in genes enriched for functions related to transcription factor activity, signal receptor binding and structural molecule expression (Fig. 1C). When compared with previously published *IRF6* siRNA human keratinocyte DEG expression data (Botti et al., 2011), there were major overlaps of genes in molecular pathways responsible for epithelial regulation, including *gata3*, *krt18* and *cldn4* (Fig. 1B,C and Figs S1-S3). Many key developmental signaling pathways including Fgf (*fgf8a*, *fgf17* and *fgf24*) and Wnt (*wnt11*, *dact2*, *rspo3*, *frzb*, *fzd5*) pathways were also heavily represented in our dataset as genes downregulated because of *irf6* ablation (Fig. 1B,C). Further, a number of genes associated with human orofacial clefts were also downregulated in the *irf6* null embryos, including *hey1*, *gata3*, *wnt11* and *fgf8* (Fig. 1B,C).

Interestingly, one of the most downregulated genes was *esrp1*. *esrp1* and its paralog *esrp2* are epithelial-restricted RNA splicing regulators. *ESRP2* genetic variants in humans are associated with orofacial clefts (Cox et al., 2018), and *Esrp1* and *Esrp1/2* knockout mice display a bilateral cleft of the lip, primary and secondary palate (Bebee et al., 2015; Lee et al., 2020). To confirm the RNA-seq results, we performed quantitative PCR (qPCR) on mz-*irf6*^-8bp/-8bp^ and WT embryos at 4-5 hpf. Relative to WT, *esrp1* expression in mz-*irf6*^-8bp/-8bp^ embryos was reduced ∼80%. Additionally, injection of mz-*irf6*^-8bp/-8bp^ embryos with *irf6* mRNA at the one-cell stage rescued *esrp1* expression, resulting in an increase in expression to ∼3-fold higher than WT (Fig. 1D). These rescued fish were phenotypically normal, as previously shown (Li et al., 2017).

We tested *Esrp1* and *Esrp2* mRNA expression in embryonic day (E)11.5 *Irf6* mutant mouse embryos (*Irf6*^R84C/R84C^) and found expression to be significantly decreased relative to that in littermate WTs (Fig. S4). Additionally, *Shh* expression was decreased in *Irf6*^R84C/R84C^ embryos (Fig. S4), consistent with a previous report of decreased *Shh* expression in *Esrp1*^−/−^ mice (Lee et al., 2020). These results in zebrafish and mouse suggest that *Esrp1* gene expression is dependent on *Irf6*, either through direct regulation or the requirement of a normal periderm.

### *irf6*, *esrp1* and *esrp2* are co-expressed in the oral epithelium of zebrafish during craniofacial development

**Fig. 2.**
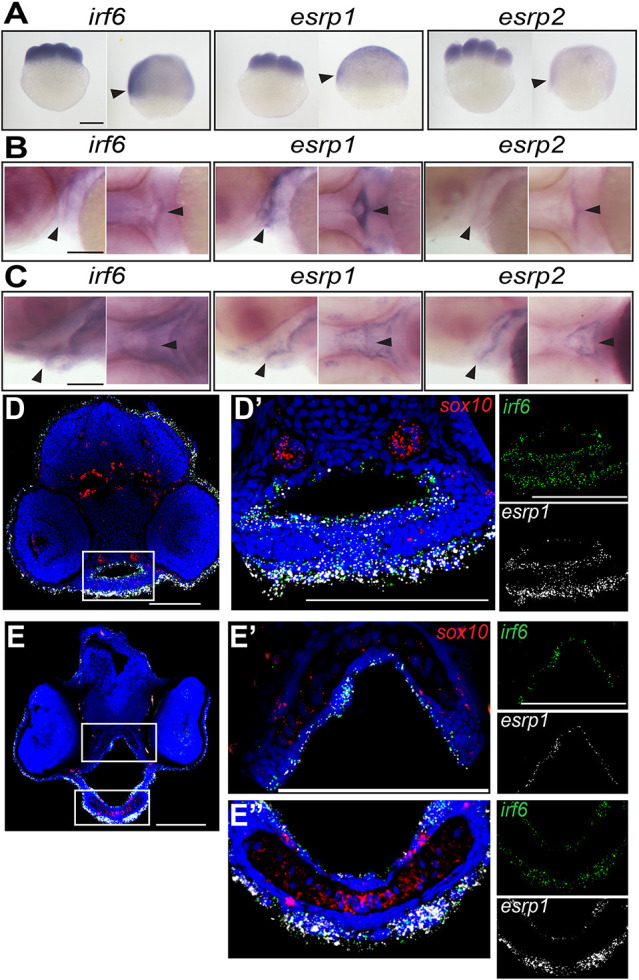
***irf6*,** ***esrp1* and *esrp2* are co-expressed in the oral epithelium of zebrafish embryos.** (A-C) Whole-mount *in situ* hybridization (WISH), showing that *irf6*, *esrp1* and *esrp2* maternal deposited transcripts are detected at the eight-cell and shield stage (A; arrowheads indicate periderm), and circumscribe the developing stomodeum and line the oral epithelium of zebrafish embryos at 48 (B) and 72 (C) hpf (arrowheads). All whole-mount embryos are oriented with anterior left and dorsal top. (D-E″) Coronal sections of 48 (D) and 72 (E) hpf embryos analyzed by RNAscope *in situ* hybridization (ISH), (dorsal top), showing cellular RNA co-expression of *irf6* (green) and *esrp1* (white) in surface and oral epithelial cells. *sox10* (red) staining depicts cartilage elements of the palate. Boxed areas are shown at higher magnification in D′, E′ and E″. Scale bars: 250 μm (A) and 100 μm (B-E″).

Previous mouse studies have described *Irf6* (Knight et al., 2006) and *Esrp1*/*2* (Bebee et al., 2015; Lee et al., 2020; Warzecha et al., 2009) gene expression in oral epithelium during palate development. To determine the gene expression of *irf6* and *esrp1/2* in the zebrafish during epithelial and craniofacial development, we performed whole-mount *in situ* hybridization (WISH). Maternal deposition of *irf6*, *esrp1* and *esrp2* mRNA was detectable at eight-cell stage (Fig. 2A). The maternal transcripts were also detected in the periderm of the gastrulating embryo, although expression of *esrp2* appeared lower than that of *irf6* and *esrp1* (Fig. 2A). During craniofacial development, WISH demonstrated specific expression of *irf6*, *esrp1* and *esrp2* lining the embryonic oral epithelium, and circumscribing surface epithelium concentrated around the developing stomodeum (Fig. 2B,C).

To resolve the specific cell populations that express *irf6*, *esrp1* and *esrp2*, we performed RNAscope *in-situ* hybridization (ISH) of coronal cryosections taken through the developing mouth and palate at 48 hpf and 72 hpf. We found that *irf6* and *esrp1* were co-expressed within epithelial cells lining the oral cavity as well as the surface epithelium (Fig. 2D,E). No expression of these genes was detected within the cartilage elements, identified by *sox10* expression. Further, we detected *irf6* and *esrp1* transcripts within the same cells, importantly within cells separating adjacent mesenchymal elements (Fig. 2D′); these cells are likely in the epithelial lineage as *esrp1* is an epithelia-specific gene.

### *Irf6*, *Esrp1* and *Esrp2* are co-expressed in murine frontonasal and oral epithelium during palate and lip development

**Fig. 3.**
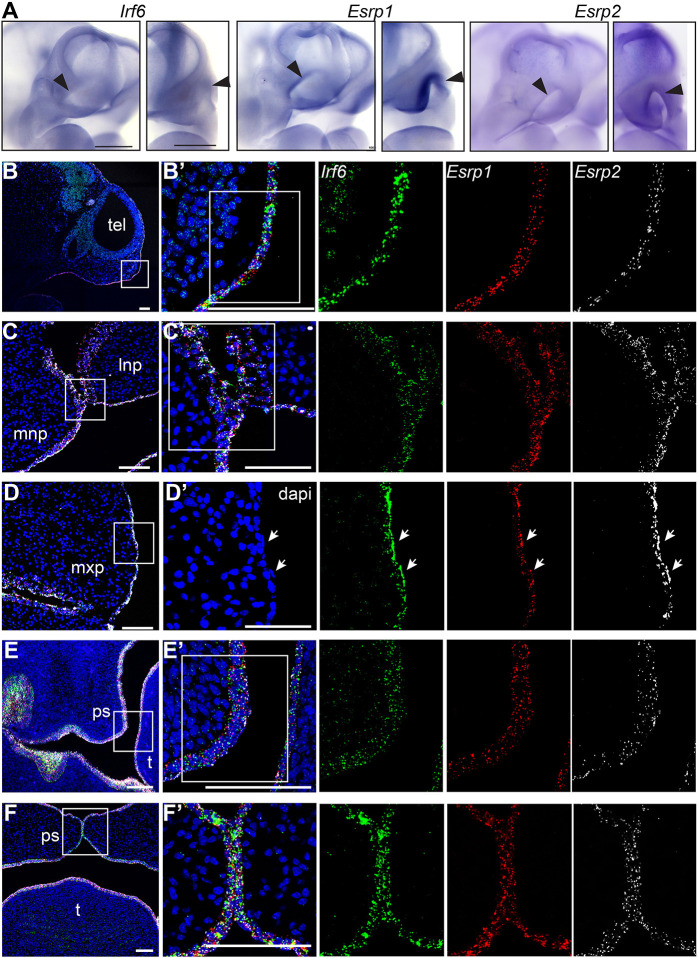
***Irf6*, *Esrp1* and *Esrp2* are co-expressed in the oral epithelium of mouse embryos.** (A) WISH of E10.5 embryos, showing *Irf6*, *Esrp1* and *Esrp2* mRNA expression in the surface epithelium and concentrated within the ectoderm of the frontonasal prominences (arrowheads) and first brachial arch. Oblique and frontal orientation. Scale bars: 500 μm. (B-F′) Sections of E10 (B,B′), E11.5 (C-D′), E13.5 (E,E′) and E15 (F,F′) embryos analyzed by RNAscope ISH, showing mRNA cellular co-expression of *Irf6* (green), *Esrp1* (red) and *Esrp2* (white) in the surface ectoderm (E10), lining the frontonasal and maxillary prominences, including expression in periderm (arrows) (E11.5), and lining the palatal shelves (E13.5, E15). Sagittal (B,B′) and coronal (C-F′) sections; boxed areas are shown at higher magnification in B′, C′, D′, E′ and F′. dapi, 4′,6-diamidino-2-phenylindole; lnp, lateral nasal prominence; mnp, medial nasal prominence; mxp, maxillary prominence; ps, palate shelf; t, tongue; tel, telencephalon. Scale bars: 100 μm.

*Irf6* expression within the embryonic oral epithelium and surrounding the developing palatal shelves has been well established (Knight et al., 2006; Iwata et al., 2013; Kousa et al., 2017). *Esrp1/2* expression was previously shown in the oral epithelium of developing mice (Bebee et al., 2016; Lee et al., 2020; Warzecha et al., 2009). Ablation of *Irf6* or *Esrp1/2* causes a cleft of the secondary palate, but the disruption of the lip and primary palate phenotypes differ between the *Irf6* and *Esrp1/2* mutants (Richardson et al., 2006; Ingraham et al., 2006; Bebee et al., 2015; Lee et al., 2020). To determine whether *Irf6*, *Esrp1* and *Esrp2* transcripts colocalize during mouse craniofacial development, we performed WISH for each gene at E10.5, as the frontonasal prominences and lambdoidal junction are taking shape at this time point. We found that *Irf6*, *Esrp1* and *Esrp2* were expressed similarly, with high levels of expression in areas of craniofacial development (Fig. 3A). The mouse gene expression pattern was similar to that observed in zebrafish, with more concentrated expression to the developing head. Higher-resolution imaging with RNAscope ISH detected *Irf6*, *Esrp1* and *Esrp2* transcripts in the periderm and the basal epithelium across all time points examined (Fig. 3B-F). *Irf6*, *Esrp1* and *Esrp2* were co-expressed in the surface ectoderm overlying the developing frontonasal prominences (Fig. 3B), a cell population with important signaling and inductive functions (Hu et al., 2003). Further, co-expression included cells at critical fusion points, specifically between the medial and lateral nasal prominences (Fig. 3C) and the palatal shelves (Fig. 3E,F). The co-expression of *Irf6* and *Esrp1*/*2* within cells with key roles during epithelial fusion supports the existence of an *Irf6-Esrp1/2* regulatory axis during craniofacial morphogenesis.

Interestingly, in addition to *Irf6* expression in the epithelium, RNAscope ISH detected *Irf6* mRNA expression in the mesenchyme, particularly at E10 and E15.5 (Fig. 3B,E). Expression of *Irf6* in this craniofacial mesenchyme has not been reported previously, and high transcript detection with RNAscope ISH might be delineating gene expression not previously observed. Non-epithelial *Irf6* expression was detected in CNCCs of the first and second pharyngeal arches at E9 (Fakhouri et al., 2017), and *Irf6* is expressed in cells of the developing tongue (Goudy et al., 2013). Further, we previously reported that zebrafish expressing the *irf6*^R84C^ variant under a *sox10* promoter exhibit a partial cleft of the ANC (Dougherty et al., 2013). Together, these results suggest an additional role of *Irf6* in craniofacial development beyond its role in epithelial cell differentiation.

### Disruption of *irf6* during neural crest cell migration results in cleft in zebrafish

**Fig. 4.**
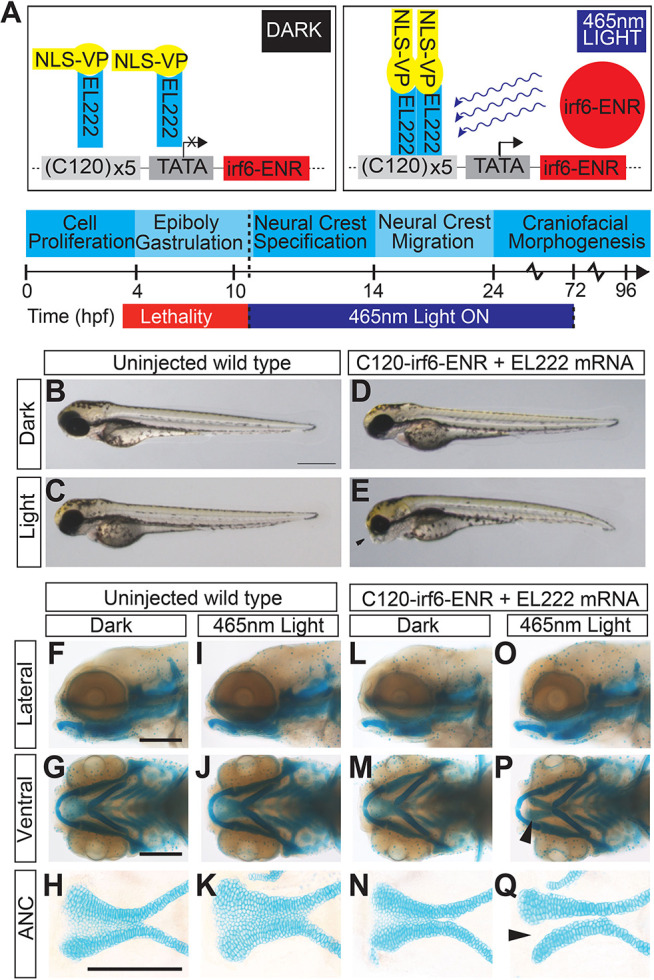
**EL222 optogenetic disruption of *irf6* circumvents early embryonic lethality and causes a cleft palate phenotype.** (A) Schematic of EL222 optogenetic system. VP16-EL222 monomers are inactive under dark conditions. Upon stimulation by 465 nm light, VP16-EL222 dimerizes, drives gene expression downstream of the C120 promoter and induces the expression of a dominant-negative form of irf6 (*irf6*-ENR). Embryos were exposed to blue light from 10 hpf to 72 hpf to circumvent embryonic lethality in mz-*irf6*^-8bp/-8bp^ embryos. (B-E) Brightfield microscopy of 72 hpf zebrafish embryos injected with the optogenetic system and grown in the dark (D) or exposed to blue light from 10-72 hpf (E) compared with control injected embryos (B,C). Injected fish exposed to blue light exhibit retrusion of the midface (arrowhead) and a curved body not observed in the other groups. (F-Q) Alcian Blue staining of cartilage and microdissection of the palate of 72 hpf embryos reveals a midface retrusion and cleft phenotype through the medial ethmoid plate (arrowhead in P, Q) in the C120-*irf6*-ENR-injected embryos grown under blue light (O-Q), which is not seen in control injected embryos (I-K) or injected embryos grown in the dark (L-N). Scale bars: 150 µm.

Germline mutation of *irf6* results in early embryonic lethality as a result of periderm rupture, which precluded evaluation of palate morphogenesis (Sabel et al., 2009; Li et al., 2017). To circumvent embryonic lethality, we employed an optogenetic gene-activation system based on the light-sensitive protein EL222, which serves to induce the expression of genes downstream of the C120 promoter (Motta-Mena et al., 2014). To this end, a dominant-negative form of *irf6* consisting of a fusion protein of the *irf6* protein-binding domain and the engrailed repressor domain (*irf6*-ENR) was cloned downstream of the C120 promoter (C120-*irf6*-ENR; Fig. 4A) (Sabel et al., 2009). When co-injected with VP-16 mRNA, this light-activated *irf6*-ENR construct enabled us to control the timing of *irf6* disruption by exposing the embryos to a 465 nm light source later in embryogenesis (Fig. 4A). Zebrafish embryos injected with the optogenetic system and continuously exposed to blue light from 10 hpf to 96 hpf were able to survive, but developed with a slightly curved body axis and a dysmorphic ventral cartilage phenotype (Fig. 4E), which were not observed in uninjected embryos (Fig. 4B,C) or injected embryos that were raised in the dark (Fig. 4D). Further analysis of the cartilage in these embryos (Fig. 4F-Q) revealed a cleft in the ANC, where a population of cells in the median portion was absent (Fig. 4Q). Moreover, injecting increasing doses of EL222-VP-16 mRNA and/or C120-*irf6*-DN (dominant negative) plasmid led to a dose-dependent effect on the proportion of zebrafish embryos with a cleft phenotype, which was more pronounced for injected embryos grown in blue light starting at 10 hpf compared with embryos grown in the dark (Fig. S5). Consistent with decreased expression of *esrp1* in mz-*irf6*^-8bp/-8bp^ embryos, disruption of *irf6* using this optogenetic system resulted in decreased expression of *esrp1* (Fig. S5).

### The compound homozygote of *esrp1* and *esrp2* exhibits cleft lip and ANC in zebrafish

**Fig. 5.**
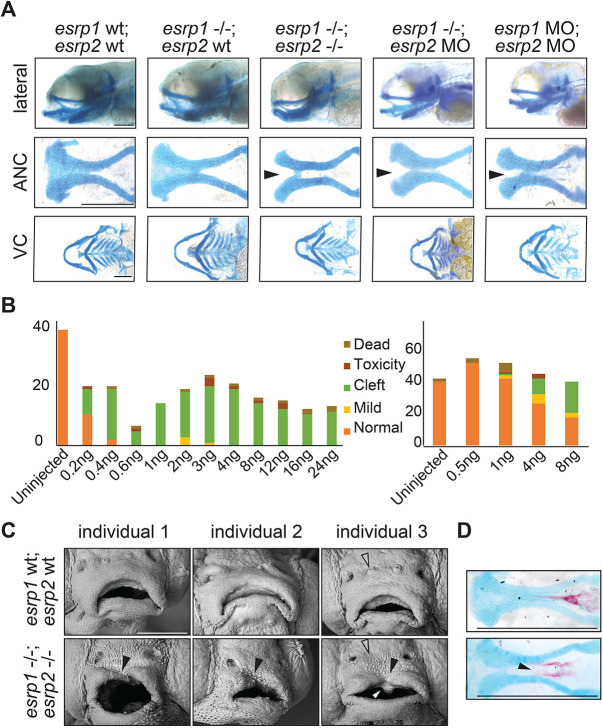
***esrp1/2* double mutants display a cleft lip and palate.** (A) Alcian Blue staining of 4 dpf zebrafish. Representative images of WT, *esrp1* CRISPR mutant (*esrp1*^−/−^) and *esrp1/2* double CRISPR mutant (*esrp1*^−/−^; *esrp2*^−/−^), as well as *esrp1* CRISPR mutant treated with *esrp2* morpholino and WT treated with *esrp1* and *esrp2* morpholino (*esrp1* MO, *esrp2* MO). Flat-mount images of the anterior neurocranium (ANC) show a cleft (arrowheads) between the median element and lateral element of the ANC when both *esrp1* and *esrp2* function were disrupted. Lateral images and flat-mount images of the ventral cartilage (VC) show only subtle changes in morphology between WT and *esrp1/2*^−/−^ zebrafish. (B) Morphant phenotypes observed over a range of *esrp1* and *esrp2* MO doses. Single *esrp2* MO injections in the *esrp1*^−/−^ background achieves nearly 100% phenotype penetrance, even at very low MO doses. (C) SEM of 5 dpf zebrafish showing discontinuous upper lip (filled arrowheads) in the *esrp1/2* double CRISPR mutant as well as absent preoptic cranial neuromasts (open arrowheads) and abnormal keratinocyte morphology. The white arrowhead indicates an aberrant cell mass. (D) Representative images of Alizarin Red/Alcian Blue staining of 9 dpf *esrp1/2* double CRISPR mutant zebrafish and WT clutch-mate controls. *Esrp1/2* ablation causes abnormal morphology of the mineralizing parasphenoid bone; the bone appears wider and with a cleft (arrowhead). Scale bars: 150 µm (A,D); 100 µm (C).

To investigate the genetic requirement for *esrp1* and *esrp2* in zebrafish craniofacial development, CRISPR/Cas9 genome editing was utilized to generate *esrp1* and *esrp2* mutant alleles. Several alleles of *esrp1* and *esrp2* were obtained, where alleles harboring −4 bp and −14 bp indels that lead to frameshift mutations and early protein truncation were selected for breeding, hereafter referred to as *esrp1*^-4bp/-4bp^ and *esrp2*^-14bp/-14bp^, respectively (Fig. S6). No phenotype was observed in the *esrp1*^-4bp/-4bp^ embryos, and *esrp2*^-14bp/-14bp^ fish developed normally (Fig. S6), except that females were infertile, as previously published in independently derived CRISPR alleles of *esrp1/2* (Burguera et al., 2017). However, compound homozygote *esrp1*^-4bp/-4bp^; *esrp2*^-14bp/-14bp^ zebrafish exhibit several phenotypes, consistent with previously published mutants (Burguera et al., 2017). The *espr1*^-4bp/-4bp^; *esrp2*^-14bp/-14bp^ embryos also failed to inflate the swim bladder, and the pectoral fins were formed but diminutive, and the margins of the fin appeared dysplastic with irregular morphology. Further, Alcian Blue staining of double knockouts revealed cleft in the ANC, whereas the ventral cartilages, including the Meckel's cartilage, were formed and appeared wild-type (Fig. 5A).

Inter-cross of *esrp1*^-4bp/-4bp^; *esrp2*^wt/-14bp^ produces predicted Mendelian ratio of 25% *esrp1*^-4bp/-4bp^; *esrp2*^-14bp/-14bp^ embryos for downstream phenotypic analysis, where 75% of the embryos appeared wild type. In order to increase the percentage of embryos that can be utilized for analysis to 100%, we asked whether morpholino (MO) disruption of *esrp2* in the *esrp1*^-4bp/-4bp^ background would consistently yield a cleft ANC phenotype that phenocopied the *esrp1*^-4bp/-4bp^; *esrp2*^-14bp/-14bp^ mutant. We successfully phenocopied the cleft ANC phenotype by co-injecting *esrp1* and *esrp2* MOs into WT embryos. However, the MO concentrations needed were relatively high, requiring 2-8 ng of each MO to be injected for ∼25-50% of embryos to develop a cleft (Fig. 5A,B). Importantly, when *esrp1*^-4bp/-4bp^ embryos were injected with *esrp2* MO, the cleft ANC phenotype was consistent and observed in nearly 100% of embryos, even when the MO concentration was reduced as low as 0.4 ng (Fig. 5A,B). One explanation for this observation is that transcriptional compensation between *esrp1* and *esrp2* occurs when each gene is targeted, thereby requiring higher doses of each MO to ablate esrp activity sufficiently (Rossi et al., 2015). But when one of the esrp genes is already disrupted in the homozygous *esrp1*^-4bp/-4bp^ mutant, the threshold for full esrp loss of function is lower, requiring a much smaller dose of MO to generate the cleft ANC phenotype.

Using scanning electron microscopy (SEM), we observed that the cleft of the upper margin of the stomodeum had invaginated and extended into the cleft of the ANC. Additionally, the keratinocyte morphology of the surface epithelium appeared irregular and round with epithelial blebs in the *esrp1*^-4bp/-4bp^; *esrp2*^-14bp/-14bp^ embryo. By contrast, the WT surface epithelium keratinocytes appeared octagonal or hexagonal without epithelial blebs (Fig. 5C). Alizarin Red staining of the larvae at 9 days post-fertilization (dpf) also revealed a lack of mineralization at the midline of the parasphenoid bone (Fig. 5D), consistent with a cleft of ANC that persisted to the ossification stage and subsequent larval development.

### Zebrafish ANC morphogenesis is dependent on epithelial interactions with infiltrating cranial neural crest cells

Formation of the zebrafish ANC involves migration of anteriormost CNCCs to populate the median portion (frontonasal derived), while more posterior CNCCs migrate from each side (maxillary derived). These three discrete embryonic elements fuse to form the ANC. Concurrent with these cellular movements, the CNCCs undergo differentiation to chondrocytes (Reid et al., 1986; Dougherty et al., 2013). We found that the ablation of *irf6* (a key periderm/epithelial gene) and *esrp1/2* (epithelial-restricted genes) both resulted in a cleft in the ANC, where chondrocytes were absent along the fusion plane between the frontonasal-derived median element and one side of the maxillary-derived lateral element (Fig. 4C and Fig. 5A).

**Fig. 6.**
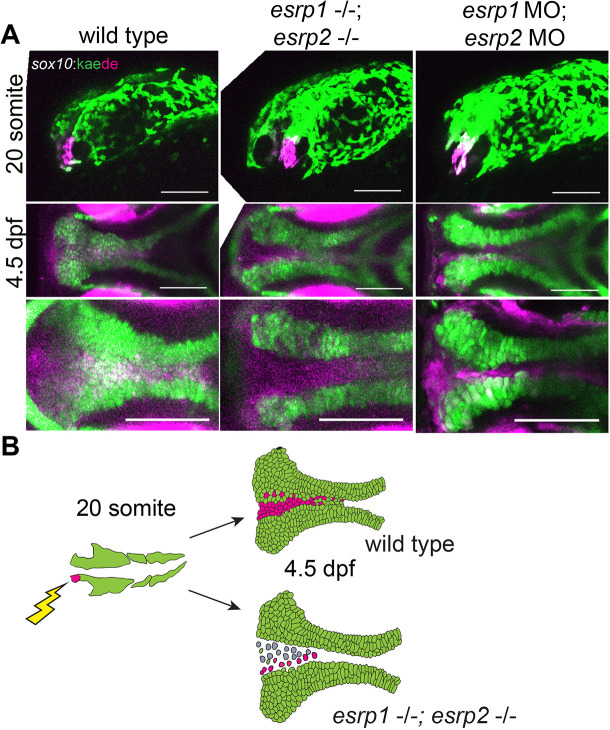
***esrp1/2* null cranial neural crest cells** **(CNCCs)** **migrate to the ANC but do not differentiate to chondrocytes.** (A) Lineage tracing of WT or *esrp1/2* morphant zebrafish embryos using the Tg(*sox10:kaede*) line, native Kaede fluorescence is shown in green, and photo-converted Kaede is shown in magenta. Sagittal and horizontal views of zebrafish embryos at 19 hpf and 4.5 dpf, respectively. The anteriormost neural crest frontonasal prominence (FNP) progenitors were photoconverted at 19 hpf. At 4.5 dpf, the WT signal tracks to the medial portion of the ANC. Both the *esrp1*/esrp2 double CRISPR mutants and *esrp1/2* morphants exhibit a cleft in the ANC with absence of a portion of *sox10*^+^ cells in the medial portion of the ANC, but the labeled CNCCs representing FNP progenitors did reach and populate the entire length of the ANC. (B) Illustrative summary of lineage tracing results showing that photo-converted anteriormost CNCCs contributing to FNP do migrate into the ANC in *esrp1/2* mutant embryos, but a cleft forms at the juxtaposition of the FNP-derived median element and the maxillary-derived lateral element. Scale bars: 150 µm.

To investigate the absence of these ANC chondrocytes, we performed lineage tracing of CNCCs in *esrp1/2*-ablated embryos. Previously, we and others identified that the anteriormost CCNC populations at 20 somites migrate to and populate the median (frontonasal) element of the ANC (Reid et al., 1986; Dougherty et al., 2013). Accordingly, we labeled the CNCCs at 20-somite stage through photo-conversion of Kaede under the lineage specificity of the *sox10* promoter. CNCCs of WT or *esrp1/2* CRISPR mutants or *esrp1/2* morphants were photo-converted at 12-15 hpf (Fig. 6A,B). Embryos were imaged at 4 dpf to determine the population of the ANC contributed by photo-converted cells. We found that *esrp1/2* ablation did not affect the ability of CNCCs to migrate into the ANC and reached posterior positions without clustering anteriorly (Fig. 6A,B). These results suggest that the cleft of the ANC in the *esrp1/2* mutants is not caused by total absence of progenitor cells or a defect in CNCC migration into the ANC. Nevertheless, Alcian Blue staining confirmed that chondrocytes were absent from a cleft in the ANC in the *esrp1/2* mutants (Fig. 5A).

**Fig. 7.**
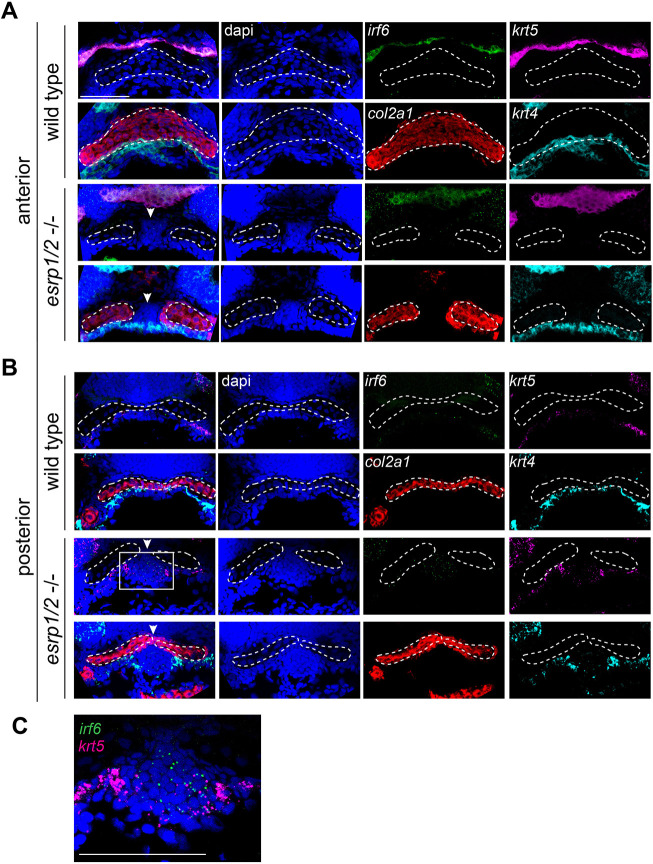
**ANC of *esrp1/2* double mutants is populated by undifferentiated cells.** Representative *z*-stacks of RNAscope ISH of coronal sections of *esrp1/2* double CRISPR mutants and WT clutch-mate controls at 4 dpf. (A) Sections through ANC anterior to the eyes. *col2a1* (red) staining depicts normal morphology of the ANC cartilage elements in WT, while a cleft is apparent in the *esrp1/2*^−/−^ zebrafish, with dapi (blue)-stained cells between adjacent trabeculae (arrowheads). These *col2a1*^–^ cells do not express epithelial markers *krt4* (cyan) or *krt5* (magenta), except around the periphery. (B) Sections posterior to those in A show *col2a1*^–^ cells continuing inferior to the trabeculae in the *esrp1/2* mutant zebrafish, and cells have low expression of *irf6* (boxed area). (C) Zoomed image of *col2a1*^–^ cells from the boxed area in B, showing *irf6* expression (green). Dashed lines outline ANC cartilage elements. Scale bars: 50 μm.

**Fig. 8.**
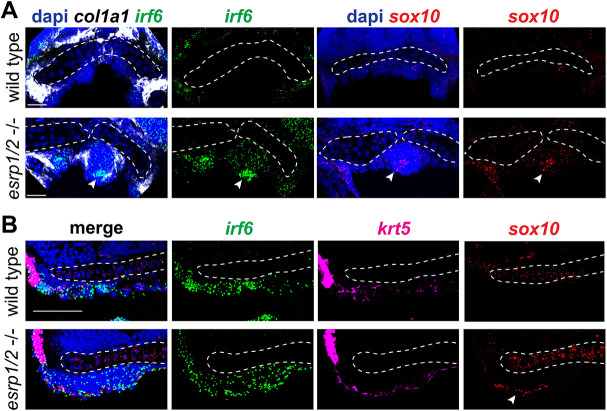
**Aberrant ANC cells of *esrp1/2* double mutants express CNCC and epithelial cell markers.** Representative *z*-stacks of RNAscope ISH of coronal sections of *esrp1/2* double CRISPR mutants and WT clutch-mate controls at 4 dpf. (A) Sections through the ANC anterior to the eyes. (B) Medial sagittal sections through the ANC (anterior to left). Dashed lines outline the ANC cartilage elements. *col1a1* (white) staining depicts perichondrium surrounding the aberrant mass of cells in the *esrp1/2* mutant zebrafish, consistent with chondrogenic condensation (leftmost arrowhead). *irf6* (green) and *sox10* (red) expression is apparent in these cells (indicated by arrowheads in respective columns); dapi is shown in blue. Scale bars: 20 μm.

To investigate the cellular composition of the ANC cleft, we performed RNAscope ISH staining of WT and *esrp1/ 2* mutants at 4 dpf. Sections through ANC clefts showed a dense population of cells in the location of the cleft (Fig. 7). In fact, this mass of cells can be localized in the SEM image of the *esrp1/2* mutant larvae (Fig. 5C). These cells are *col2a1*^–^, consistent with absent Alcian Blue staining. Instead, this aberrant cell population expresses *irf6*, while *krt4* and *krt5* staining is restricted to the periphery, consistent with the epithelial lining of the oral cavity (Figs 7 and 8). Coronal and sagittal sectioning through the medial ANC of WT and *esrp1/2* mutant embryos confirmed the ectopic expression of *irf6* and revealed *sox10* expression in these aberrant, Alcian Blue^–^ cells (Fig. 8A,B). Like *krt4*, the expression of *krt5* outlines the oral cavity (Fig. 8B). The expression of *sox10* suggests that at least a portion of these cells was CNC derived, whereas *krt4* expression indicates an epithelial lineage. The presence of *irf6* expression could be indicative of epithelial/periderm cells, or indicative of expression by CNCCs, as has previously been reported (Dougherty et al., 2013; Kousa et al., 2019). Based on these results, we hypothesize that epithelial (and/or periderm) cells associated with frontonasal and maxillary prominence derivatives are defective in the *esrp1/esrp2* null mutants, and either disrupt or fail to promote fusion of the median and lateral elements of the ANC, causing a cleft to form (Fig. 10). In this way, this is the first direct evidence of cleft pathogenesis in the zebrafish as a result of epithelial defect, and suggests a model to consider how cleft pathogenesis involving the primary palate is conserved across vertebrates (Iwata et al., 2013; Ingraham et al., 2006; Bebee et al., 2015; Richardson et al., 2006).

### Genetic interaction of *Irf6*^R84C^ with *Esrp1* and *Esrp2*

To test the hypothesis that *Irf6* and *Esrp1/2* genes function in the same developmental pathway, we carried out genetic epistasis analysis and generated *Irf6*; *Esrp1*; *Esrp2* compound mutants. We hypothesized that if *Irf6* and *Esrp1/2* genetically interact, then *Irf6* and *Esrp1* heterozygosity on an *Esrp2* null background might result in a cleft phenotype, when *Irf6* and *Esrp1* heterozygotes do not normally form a cleft. As expected, we observed that *Irf6*^R84C/+^; *Esrp1*^+/−^; *Esrp2*^+/−^ mice developed and reproduced normally. To generate *Irf6*^R84C/+^; *Esrp1*^+/−^; *Esrp2*^−/−^ embryos, we intercrossed the triple heterozygous mice. We collected a total of 79 embryos from nine litters from E12.5 to E18.5 and tabulated the resulting genotypes (Table 1, Table S2). Based on Mendelian genetics, we expected approximately five *Irf6*^R84C/R84C^; *Esrp*1^–/–^ double homozygous mice. However, these breedings did not produce any *Irf6*^R84C/R84C^; *Esrp1*^−/–^ embryos (Table 1). This result suggested that compound ablation of *Irf6* and *Esrp1* is more deleterious to development than either genotype alone, and supports a genetic interaction between *Irf6* and *Esrp1*, which could be essential early in development.

**Table 1.**
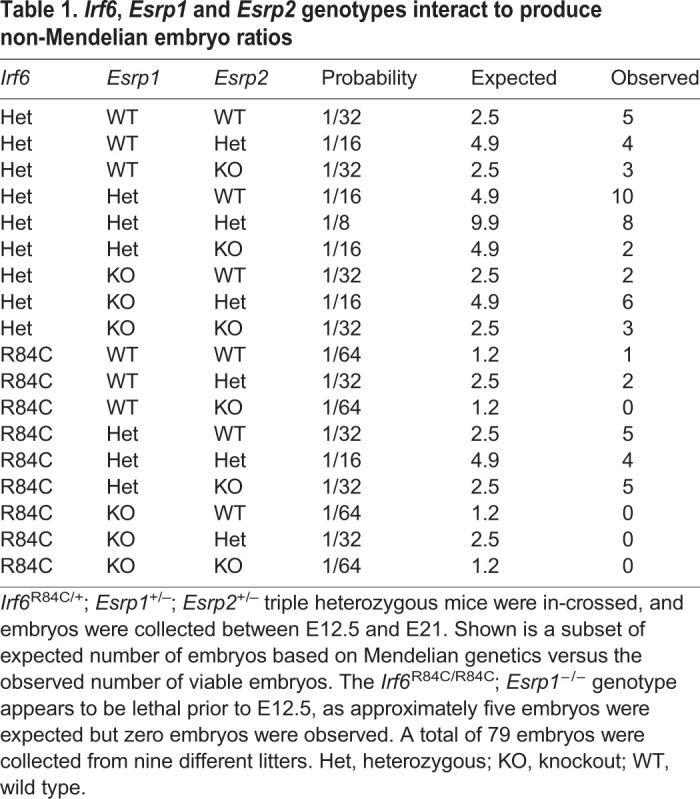
***Irf6*, *Esrp1* and *Esrp2* genotypes interact to produce non-Mendelian embryo ratios**

**Fig. 9.**
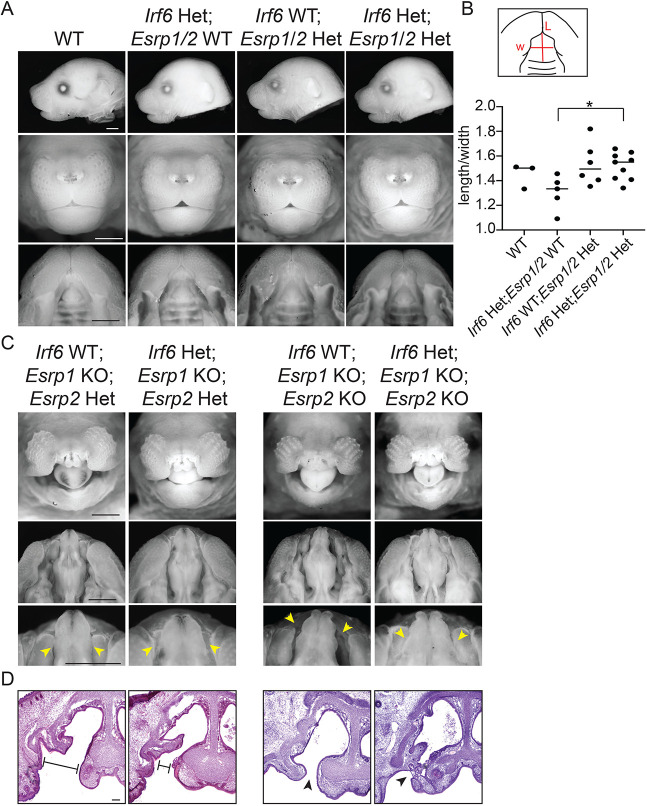
***Irf6* and *Esrp1/2* interact to modify palate phenotypes.** Mice compound heterozygous for *Irf6*^R84C^, *Esrp1* and *Esrp2* were generated by breeding *Irf6*^R84C*/*+^ with *Esrp1^+/−^*; *Esrp2*^−/−^ mice. The triple heterozygotes were then inter-crossed and embryos were collected at E18.5. (A) Representative lateral, frontal and oral images of embryos, comparing WT (*Irf6^+^*^/+^; *Esrp1*^+/+^; *Esrp2*^+/+)^, *Irf6*^R84C^ heterozygote (Het) (*Irf6*^R84C/+^; *Esrp1*^+/+^; *Esrp2*^+/+^), *Esrp1/2* double heterozygote (*Irf6*^+/+^; *Esrp1*^+/−^; *Esrp2*^+/−^) and triple heterozygote (*Irf6*^R84C/+^; *Esrp1*^+/−^; *Esrp2*^+/−^). (B) Measurements of palate length (L) relative to width (W). *Irf6*^R84C/+^ embryos tend to have a shorter palate compared with WT; however this genotype on an *Esrp1*^+/−^; *Esrp2*^+/−^ background results in significantly increased palate length relative to *Irf6*^R84C/+^; *Esrp1*^+/+^; *Esrp2*^+/+^ (one-way ANOVA, *P<0.05; *n*=3,5,6,9). (C) Representative frontal and oral images of embryos, comparing *Irf6*^+/+^; *Esrp1*^−/−^; *Esrp2*^+/−^ with *Irf6*^R84C^^/+^; *Esrp1*^−/−^; *Esrp2*^+/−^ and *Irf6*^+/+^; *Esrp1*^−/−^; *Esrp2^−^*^/−^ with *Irf6*^R84C/+^; *Esrp1*^−/−^; *Esrp2*^−/−^. Scale bars: 50 μm. (D) Hematoxylin and Eosin staining of coronal sections through the vomeronasal cavity and primary palate of the same embryos. *Irf6*^R84C^ heterozygosity modifies the *Esrp1* knockout (KO) and *Esrp1/2* double KO cleft lip and palate such that the cleft space between adjacent elements is narrower (arrowheads; C,D), and, in some cases, we noticed epithelial adhesions that limited the cleft. Scale bars: 100 μm.

To test for phenotypic effects in the resulting *Irf6*; *Esrp1*; *Esrp2* compound mutants, we imaged embryos at E18.5. We did not observe any cleft lip or palate in any genotype collected except for the expected clefts when null for *Irf6* (Fig. S7) or *Esrp1* (Fig. 9). *Irf6*^R84C/+^; *Esrp1*^+/−^; *Esrp2*^−/−^ embryos that we predicted to be susceptible to cleft lip and/or palate were grossly normal (Fig. S7). We noticed some differences in the shape of the palate between heterozygous genotypes and measured the length (from philtrum to first rugae) relative to the width (space between lips). We found that *Irf6*^R84C/+^ heterozygotes exhibited a shorter palate than WT (Fig. 9A,B). A shorter snout has previously been reported in *Irf6* KO mice (Ingraham et al., 2006). The length/width ratio of *Esrp1/2* double heterozygotes was similar to WT, and in the *Irf6*; *Esrp1*; *Esrp2* triple heterozygote, the shorter palate phenotype of the *Irf6* heterozygote was reversed (Fig. 9A,B). Taken together, these breeding and morphologic analyses suggest an overlapping role of *Irf6* and *Esrp1/2* in regulating midface morphogenesis.

**Fig. 10.**
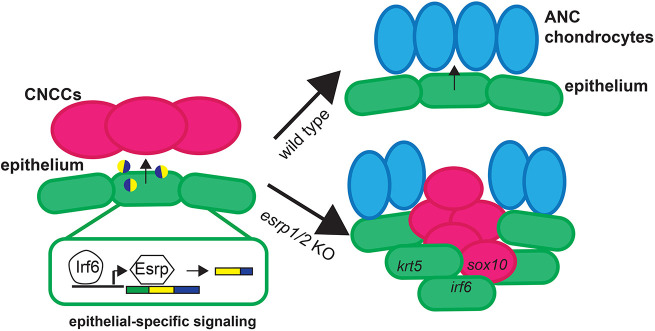
**Illustrative summary of results.** Ablation of the epithelial-restricted splicing factors *esrp1* and *esrp2* led to the dysregulation of CNCC integration and differentiation in the medial ANC, causing a cleft between lateral ANC elements. These results suggest that epithelial-specific splice variants of yet to be determined factors are required for directing the juxtaposed mesenchymal-derived cells and promoting normal morphogenesis.

As previously reported, *Esrp1* null and *Esrp1/2* double null embryos displayed bilateral CL/P (Fig. 9C). Interestingly, we noted a modification of this cleft phenotype when *Esrp1* and *Esrp1/2* null embryos were also heterozygous for *Irf6*. *Irf6*^R84C/+^; *Esrp1*^−/−^; *Esrp2*^+/−^ and *Irf6*^R84C/+^; *Esrp1*^−/−^; *Esrp2^−^*^/−^ embryos had less space or less wide clefts between the lateral lips and maxilla and the midline nasal capsule (Fig. 9C). This decreased space between tissue (or cleft severity) but persistence of a cleft was confirmed in histological sections (Fig. 9D). Further, histological sections showed presumed epithelial adhesions between lateral and medial portions of the nasal cavity in *Irf6* heterozygotes, whereas this space was open in *Esrp1* null and *Esrp1/2* double null embryos (Fig. 9D).

## DISCUSSION

Orofacial clefts are a common birth defect, and genome-wide association studies have identified some crucial genes associated with syndromic and non-syndromic cleft. Here, we describe mouse and zebrafish models using genes with known genetic variants in human cleft patients, *IRF6* and *ESRP1/2*. We present evidence to support that *Irf6* and *Esrp1/2* function in the same regulatory pathway. We observed that mz-*irf6*^-8bp/-8bp^ zebrafish embryos have significantly decreased expression of *esrp1*, and this is rescued upon introduction of *irf6* mRNA. This finding is consistent with *esrp1* being a transcriptional target of *irf6*, and putative *irf6* response elements (Khan et al., 2018) can be found surrounding the *esrp1* transcriptional start site. Additionally, RNA-seq identified known *irf6* targets, including *grhl3* and *tfap2a*. Direct molecular experiments are needed, however, to test transcriptional regulation of *esrp1* by *irf6*. We found that *Irf6* and *Esrp1/2* are consistently co-expressed in the embryonic frontonasal ectoderm and oral epithelium associated with the palate, and epithelium of the mouth opening, in both mouse and zebrafish.

In zebrafish, *irf6* null embryos ruptured during gastrulation, whereas *esrp1/2* null embryos survive to larval stage. However, post-gastrulation ablation of *irf6* resulted in a similar cleft morphology of the ANC as the *esrp1/2* null. Further analysis of the *esrp1/2* null showed that the cleft of the ANC correlated with a cleft in the upper margin of the mouth opening, reminiscent of a human cleft lip. Further, using a neural crest-specific photo-convertible reporter line, we were able to show that migration of CNCCs to the developing ANC occurred but chondrogenesis was impaired.

The early lethality of *irf6* null zebrafish initially precluded analysis of the *irf6* zygotic requirement in craniofacial development. Here, we utilized an optogenetics strategy to disrupt *irf6* function after gastrulation when the embryonic body axis had formed, thereby revealing the zygotic requirement for *irf6*. Future studies will use the *irf6* optogenetic model to study the roles of *irf6* during ANC and lip morphogenesis. Interestingly, periderm markers identified in the mouse lambdoidal junction were found to be dysregulated in the *irf6* mutant zebrafish model, specifically *grhl3*, *tfap2a* and *perp*. Additionally, *gata3*, which was identified as a mesenchymal marker at the fusion zone of mice (Li et al., 2019), is dysregulated in the *irf6* null zebrafish. This work highlights the utility of complementary studies of palate morphogenesis in zebrafish and mouse models. The zebrafish model affords the transgenic tractability and visualization of CNCC migration, enabling us to determine the cellular mechanism responsible for the cleft ANC. The mouse mutants provide the mammalian anatomic contexts to examine cleft malformation.

Although the *esrp1*^-4bp/-4bp^; *esrp2*^-14bp/-14bp^ zebrafish exhibited consistent cleft lip and cleft ANC phenotype, the infertility of the *esrp2*^-14bp/-14bp^ fish preclude large-scale experiments to analyze downstream mechanisms of the development of cleft palate. We generated a robust *esrp1*^-4bp/-4bp^; *esrp2* morphant assay that can be applied in chemical screening experiments and functional testing of human *ESRP1/2* gene variants.

In humans, CPO is less common than CL/P (Gritli-Linde, 2008; Van Otterloo et al., 2016; Bush and Jiang, 2012). Although humans and mice share ∼99% of their genes and the early craniofacial development of the mouse embryo closely mirrors that of human (Swartz et al., 2011), there is a striking difference in the manifestation of orofacial cleft defects (Gritli-Linde, 2008). Most often, when a human CL/P-associated gene has been disrupted in mice, a cleft of the palate forms but the lip appears normal. Our current understanding in humans is that CL/P and CPO are different genetic disorders (Gritli-Linde, 2008; Juriloff and Harris, 2008; Dixon et al., 2011). These discrepancies between humans and mouse models hamper understanding of the etiopathogenesis of human CL/P. Here, we characterize the *Esrp*1/2 null mouse, exhibiting bilateral CL/P, as an important model for studying orofacial cleft etiopathogenesis. Additionally, as we place *ESRP1* in the *IRF6* gene-regulatory pathway, we hope to better understand how alternative isoforms regulated by *ESRP1* may, in turn, be important for palate development.

Whereas zebrafish have historically been an excellent model organism for forward genetic screens, CRISPR/Cas9 gene-editing technology has permitted relatively efficient reverse genetic engineering of zebrafish (Liu et al., 2019). This utility of the zebrafish embryo for studying developmental processes and modeling human cleft-associated genes necessitates further study into their craniofacial morphogenesis. Transplant and lineage-tracing experiments have illuminated the neural crest origin of the zebrafish ANC, and how the frontonasal and paired maxillary cartilage elements converge into a continuous cartilage structure (Reid et al., 1986; Dougherty et al., 2012; Dougherty et al., 2013). We show that *IRF6* and *ESRP1* are conserved in their requirement for ANC morphogenesis, where disruption results in orofacial cleft in human, mouse and zebrafish. These findings provide evidence of conserved molecular and morphological processes occurring in the merging and fusion of the mouse and zebrafish midface.

We suspect that non-epithelial expression of *Irf6* contributes to normal craniofacial morphogenesis and may explain some differences in the *Irf6* and *Esrp1/2* mutant phenotypes. Future research utilizing tissue-specific knockout of *Irf6* will address this hypothesis. We also suspect that the *Irf6* phenotype is more severe because *Irf6* acts upstream of *Esrp1*, along with additional targets, and ongoing experiments on the transcriptional activity of *Irf6* will be important. Recently, an in-depth analysis of a lineage-specific *Esrp1* knockout mouse was completed and found that *Esrp1* regulates proliferation of the mesenchyme of the lateral nasal prominences, along with being required for fusion of the medial and lateral nasal prominences (Lee et al., 2020). Ongoing work to identify *Esrp1/2* molecular targets and mechanistic studies of these targets will provide new insight into palate morphogenesis.

These studies highlight the utility of complementary mouse and zebrafish models to elucidate mechanisms of orofacial cleft development. Additionally, this work has expanded the scope of *Irf6* gene regulation in craniofacial development.

## MATERIALS AND METHODS

### Animal breeding and gene editing

All animal experiments were performed in accordance with protocols approved by Massachusetts General Hospital Animal Care and Usage Committee. C57Bl/6J (WT) animals were obtained from The Jackson Laboratory. *Irf6*^R84C/+^ mice were a gift from Dr Yang Chai (University of Southern California, Los Angeles, USA). *Esrp1^+/–^*; *Esrp2^−/−^* mice were received from Dr Russ Carstens (University of Pennsylvania, Philadelphia, USA). E0.5 was considered to be 12:00 on the day of the copulatory plug.

Zebrafish (*Danio rerio*) adults and embryos were maintained in accordance with approved institutional protocols at Massachusetts General Hospital. Embryos were raised at 28.5°C in E3 medium (5.0 mM NaCl, 0.17 mM KCl, 0.33 mM CaCl_2_, 0.33 mM MgSO_4_) with 0.0001% Methylene Blue. Embryos were staged according to standardized developmental timepoints by hpf or dpf (Liu et al., 2001). All zebrafish lines used for experimentation were generated from the Tübingen strain.

CRISPR sgRNA target sites were identified by a variety of online CRISPR computational programs such as ZiFiT Targeter Version 4.2 (zifit.partners.org/ZiFiT) (Sander et al., 2007), crispr.mit.edu (https://zlab.bio/guide-design-resources) (Ran et al., 2013) and ChopChop (https://chopchop.cbu.uib.no) (Montague et al., 2014). sgRNAs were designed with the traditional sequence constraint of a 3′ protospacer adjacent motif sequence containing NGG and an additional sequence constraint of a 5′ NG for *in vitro* RNA synthesis.

The *esrp1*, *esrp2* and *irf6* CRISPR sgRNAs were generated by *in vitro* transcription from an SP6 promoter as described (Gagnon et al., 2014). Lyophilized Cas9 protein (PNA Bio) was resuspended in ddH_2_O to a stock concentration of 1 µg/µl and stored in single-use aliquots in −80°C and kept for 6 months. One-cell-staged zebrafish embryos were microinjected directly in the cytoplasm with 2 nl of a solution containing 15 ng/µl sgRNA and 100 ng/µl Cas9 protein pre-complexed for 5-10 min at room temperature (RT). A subset of embryos injected with the sgRNA and Cas9 protein mixture was harvested for genomic DNA to confirm the presence of indels, and the rest were grown into adulthood as F0 mosaic fish. F0 adult fish were subsequently outcrossed with WT fish to generate F1 founders with germline transmission of indel alleles. F1 founders were further outcrossed with WT fish to yield a large number of heterozygotes and minimize the presence of off-target edits. Lastly, F2 heterozygotes were in-crossed to generate homozygote embryos for phenotypic analysis.

DNA for genotyping was isolated from either whole 24 hpf embryos or tail fin clips using the HotSHOT method as previously described (Meeker et al., 2007). Genotyping primers flanking the CRISPR sgRNA site were designed using a combination of ChopChop (https://chopchop.cbu.uib.no) and NCBI primer BLAST (ncbi.nlm.nih.gov/tools/primer-blast/). Forward primers were synthesized by Invitrogen with 5′-FAM modifications. Microsatellite sequencing analyses were used to determine indel mutation sizes and frequencies (Massachusetts General Hospital DNA Core), and Sanger sequencing was performed on PCR amplicons of CRISPR sgRNA to confirm the exact sequence changes resulting from CRISPR mutagenesis.

### mRNA sequencing and qPCR

Total RNA was isolated from 4 hpf WT and maternal-null *irf6^−/−^* embryos by TRIzol and phenol-chloroform ethanol precipitation. Total RNA was quantified with the Nanodrop 2500 and assessed for quality with Bioanalyzer 2100 RNA chips (Agilent). Samples with RNA integrity numbers (RIN) over 9 were selected to proceed with sequencing library preparation. mRNA sequencing (mRNA-seq) libraries were prepared with the NEBNext Ultra RNA library preparation kit with poly(A) mRNA magnetic isolation module (NEB) essentially according to manufacturer protocols. Resulting cDNA libraries were quantified by a Qubit fluorometer and assessed for quality with a Bioanalyzer. The sequencing-ready cDNA libraries were quantified with the NEBNext library quantification kit for Illumina (NEB). mRNA-seq libraries were sequenced with single-end 50 at ≈20 million reads per sample with biological triplicates. Sequencing data are available at Gene Expression Omnibus (GEO; accession number GSE153828).

For qPCR, ∼30 zebrafish embryos per sample were flash frozen in liquid nitrogen. Mouse embryos from E11.5 timed pregnancies were isolated and dissected so that the head portion was flash frozen for RNA isolation and a posterior portion was frozen for genotyping. Samples were homogenized using a rotor-stator homogenizer, and RNA was isolated using an RNeasy Mini Kit (Qiagen). Total mRNA was quantified using a Nanodrop spectrophotometer (Thermo Fisher Scientific) and used for cDNA synthesis. qPCR was performed with Taqman probes and reagents (Thermo Fisher Scientific), and expression was normalized to 18s rRNA or *TBP* expression.

### Zebrafish embryo microinjection of mRNA and MOs

Microinjection of mRNA was performed by injecting 2 nl mRNA solution with 0.05% Phenol Red directly into the cytoplasm of one-cell-staged embryos. Lyophilized MOs were resuspended with ddH_2_O to a stock concentration of 20 ng/µl and stored at RT in aliquots. Individual aliquots were heated to 70°C and briefly vortexed before preparation of the injection mix to ensure full dissolution. Mismatch control MOs were injected under identical conditions to control for potential toxicities. Embryos from all methods of microinjection were examined at 3 hpf to remove unfertilized embryos, which were quantified against the total number of microinjected embryos to ensure that no fertilization defects were observed.

### WISH

Embryos were isolated at various time points and fixed in 4% formaldehyde at 4°C for 12-16 h. Subsequently, embryos were washed and stored in methanol. WISH- and digoxigenin (DIG)-labeled riboprobes were synthesized as described (Thisse and Thisse, 2008). Briefly, for riboprobe synthesis, PCR was performed using embryonic cDNA as templates and T7 promoter sequence-linked reverse primers to generate cDNA templates for *in vitro* transcription. PCR reactions were purified using the NucleoSpin gel and PCR clean-up kit (Machery-Nagel). *In vitro* transcription was performed using a T7 polymerase (Roche) and DIG labeling mix (Roche). DIG-labeled riboprobes were isolated with ethanol-NaOAc precipitation, resuspended in diethyl pyrocarbonate-treated ddH_2_O and stored at −20°C. All PCR products were TOPO cloned into pGEM-T Easy vectors (Promega) and sequence verified by Sanger sequencing. WISH colorimetric signal detection was performed using an alkaline phosphatase (AP)-conjugated anti-DIG antibody (Roche) and BM Purple AP substrate (Roche).

### RNAscope ISH

Zebrafish and mouse embryos were fixed in 4% formaldehyde, taken through a sucrose gradient and cryo-embedded and sectioned. Probes were designed and purchased from ACD Bio, and hybridization and staining were performed according to the manufacturer's protocol. Stained sections were imaged using a Leica SP8 confocal microscope, where a *z*-stack was obtained and analyzed on ImageJ for *z*-stack maximum-intensity projections. In cases where a larger field was imaged, Leica LAS X software was utilized to perform a tile scan and to reconstruct the tiled images.

### Skeletal staining and brightfield imaging

Zebrafish embryos were fixed at 96 hpf or 120 hpf in 4% formaldehyde and stored at 4°C overnight, washed with PBS, dehydrated in 50% ethanol, and stained with acid-free Alcian Blue overnight on a rotating platform at RT as described (Thisse and Thisse, 2008). Stained embryos were washed with ddH_2_O and subsequently bleached (0.8% w/v KOH, 0.1% Tween 20, 0.9% H_2_O_2_) until cell pigmentation was no longer present. For double-stained embryos (Alcian Blue and Alizarin Red), embryos were stained with a 0.05% Alizarin Red solution in ddH_2_O for 30 min on a rotating platform at RT following bleaching with KOH and H_2_O_2_. Then, double-stained embryos were placed in three changes of a tissue-clearing solution consisting of 25% glycerol and 0.1% KOH, each for 25 min. Whole and dissected stained embryos were mounted in 3% methylcellulose on a depression slide and imaged using a Nikon Eclipse 80i compound microscope with a Nikon DS Ri1 camera. *Z*-stacked images were taken to increase the depth of field with NIS Element BR 3.2 software. Stacked images were processed by ImageJ to generate maximum-intensity projection images.

For SEM, 4 dpf embryos were fixed in half-strength Karnovsky fixative. Samples were processed, and images were obtained by CBSET (Lexington, MA, USA). Mouse embryos from *Irf6*^R84C/+^; *Esrp1*^+/−^; *Esrp2*^+/−^ crosses were collected into PBS. Tail clips were saved for genotyping and embryos were fixed in 10% formalin for brightfield imaging. After imaging, skulls (excluding the lower jaw) were cryosectioned and sections were stained with Hematoxylin and Eosin.

### Optogenetic expression of *irf6* in zebrafish

Genes *irf6*, *irf6*-ENR, *irf6*^R84C^ and mCherry were isolated by PCR from various templates and inserted into the pGL4.23-(C120×5)-TATA vector with In-Fusion cloning (Clontech) according to the manufacturer’s instructions using a 1:2 vector-to-insert ratio to generate optogenetic response plasmids. The constructs were transformed in Stellar chemically competent cells (Clontech), and colonies were screened by PCR, restriction digests, Sanger sequencing and whole-plasmid sequencing to verify the sequence identities and accuracy of the constructs. Light-sensitive response protein VP16-EL222 was subcloned into pCS2+8 and *in vitro* transcribed from the SP6 promoter as described above to generate capped mRNA for embryo microinjections. The optogenetics injection mix consisted of 25 ng/µl EL222 and 10 ng/µl pGL4.23 response plasmid with 0.05% Phenol Red. Each embryo was microinjected with 2 nl of the optogenetics injection mix directly in the cytoplasm at the one-cell stage, immediately wrapped in aluminum foil, and placed into a dark incubator. Unfertilized and abnormal embryos were removed at 3 hpf in a dark room with limited exposure to ambient light. Injected embryos were divided into two groups (dark and light) at the desired developmental stage in E3 medium without Methylene Blue and placed under 465 nm blue light (LED panel, HQRP) at 0.3 mW/cm^2^ (measured by a PM100D digital power meter with an SV120VC photodiode power sensor, ThorLabs) with constant illumination. Control embryo containers were wrapped in aluminum foil.

### Lineage tracing

Embryos originating from an *espr1*^-4bp/-4bp^;*esrp2*^+/-14bp^ in-cross were injected with 8 ng *esrp1* MO and 4 ng *esrp2* MO at the one-cell stage, or uninjected WT embryos, all in a Tg(*sox10:kaede*) background, were grown until 20 somites, oriented for imaging in the sagittal position, and encased in 1% low-melt agarose. Using the 405 nm UV laser and ROI setting in a Leica SP8 confocal microscope, the anteriormost portion of neural crest cells that contribute to the FNP were unilaterally photoconverted, keeping the alternate side as an internal control, as previously described (Dougherty et al., 2012). Photoconverted embryos were carefully micro-dissected out of the agar and grown in E3 medium at 28.5°C until 4 dpf and imaged again to track the photoconverted cells. Maximum projections of the photoconverted half of the embryo, or the planes consisting of the palate, in 14 hpf or 4 dpf embryos, respectively, were generated using Fiji/ImageJ.

### Statistics

Statistical analyses were performed using Prism Software (GraphPad). An unpaired Student's *t*-test or one-way ANOVA with multiple comparisons was used as indicated. A *P*-value <0.05 was considered significant. Graphs represent the mean±s.e.m. or individual values (dots). In all experiments, *n* represents biological replicates.

## Supplementary Material

Supplementary information

Reviewer comments
