# Supplementary material for: An Irf6-Esrp1/2 regulatory axis controls midface morphogenesis in vertebrates
Source: Development. 2020 Dec 23;147(24):dev194498. doi: 10.1242/dev.194498 (PMC7774891; doi:10.1242/dev.194498)
Supplement: Supplementary information [file develop-147-194498-s1.pdf]

Figure S1

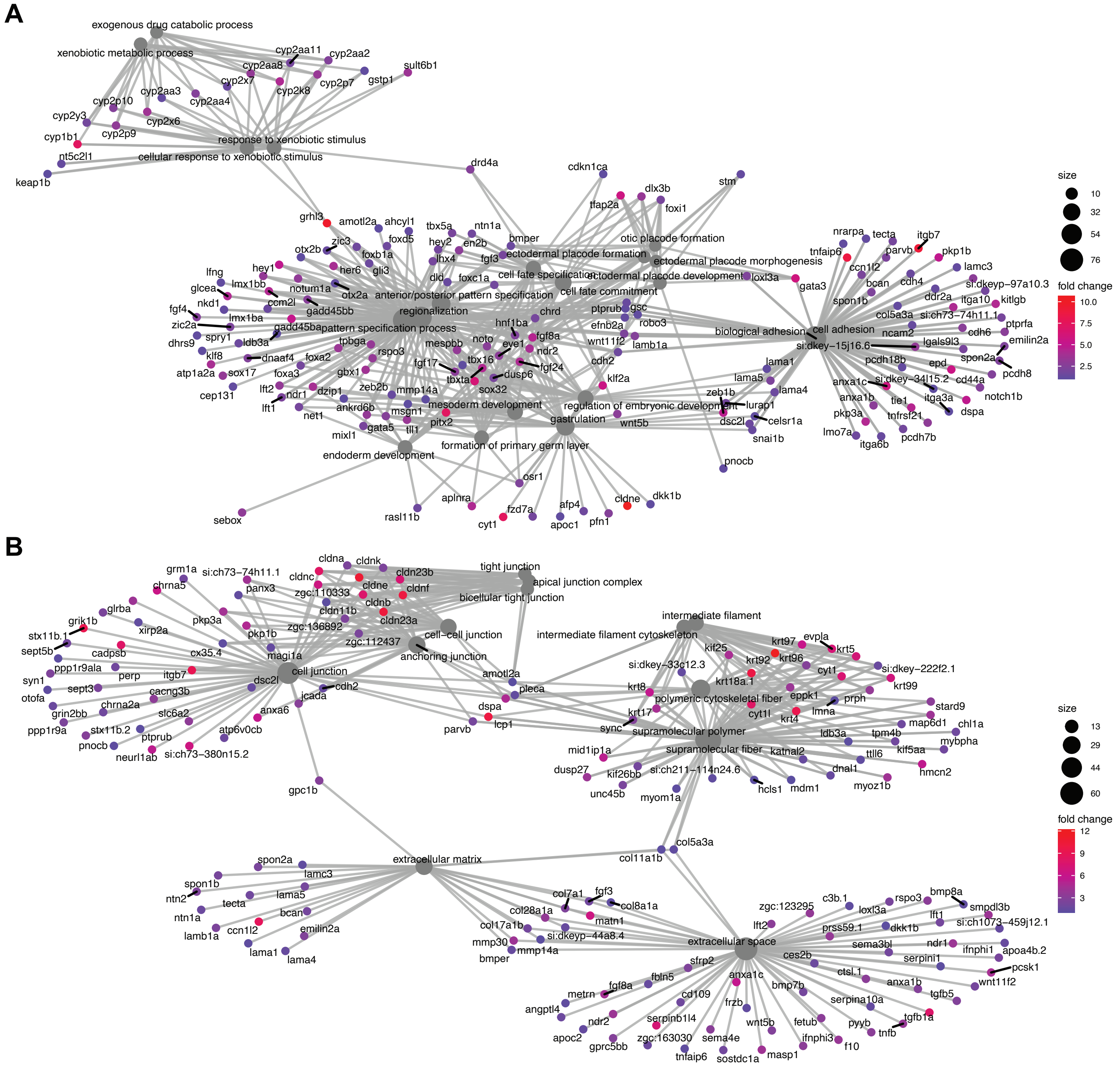

Development • Supplementary information

Figure S1. Gene ontology (GO) gene-concept network analysis of RNA-seq data. (A) *irf6*-/- embryos have perturbations in biological processes such as cell adhesion, gastrulation, mesoderm, ectoderm and endoderm development, and response to xenobiotic stimulus. (B) *irf6*-/- embryos also have perturbations of various cellular compartments including cell junctions, cytoskeletal elements and extracellular space. Grey nodes show GO terms, colored nodes show individual genes from the RNA-seq dataset, and edges connect genes to one or more associated GO terms. Colored nodes show relative enrichment (measured by fold-change) of genes in wild type samples relative to *irf6-/-* embryos. Maps were generated using the enrichplot package in R.

Figure S2

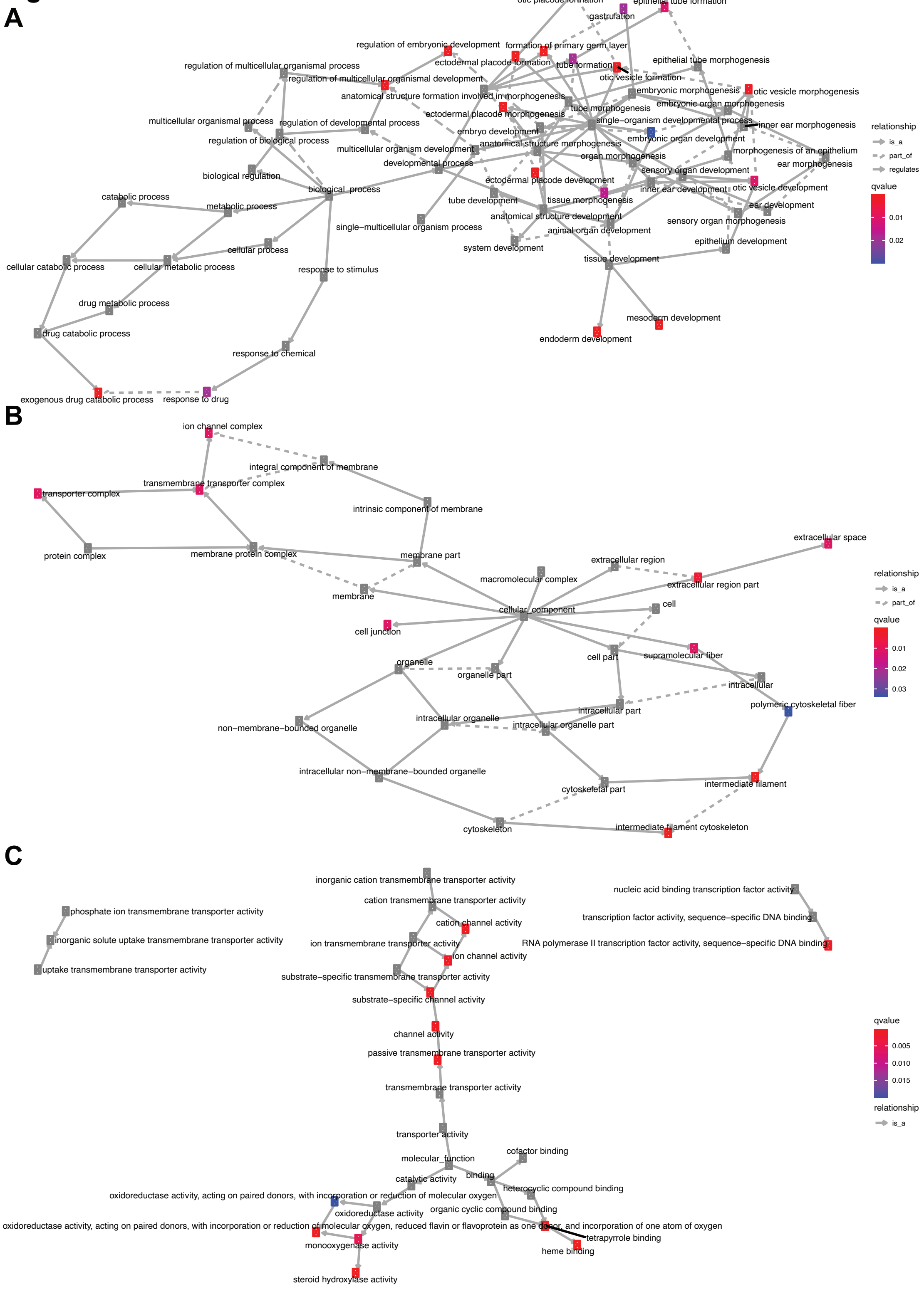
A
otic placode formation
epithelial tube formation
gastrulation
regulation of embryonic development
formation of primary germ layer
ectodermal placode formation
tube formation
epithelial tube morphogenesis
regulation of multicellular organismal process
regulation of multicellular organismal development
otic vesicle formation
embryonic morphogenesis
otic vesicle morphogenesis
anatomical structure formation involved in morphogenesis
embryonic organ morphogenesis
ectodermal placode morphogenesis
tube morphogenesis
single−organism developmental process
inner ear morphogenesis
multicellular organismal process
regulation of developmental process
embryo development
embryonic organ development
regulation of biological process
anatomical structure morphogenesis
multicellular organism development
morphogenesis of an epithelium
organ morphogenesis
ear morphogenesis
developmental process
sensory organ development
biological regulation
ectodermal placode development
biological_process
inner ear development
otic vesicle development
catabolic process
tube development
tissue morphogenesis
ear development
metabolic process
anatomical structure development
sensory organ morphogenesis
single−multicellular organism process
animal organ development
cellular process
epithelium development
system development
cellular catabolic process
cellular metabolic process
tissue development
response to stimulus
drug metabolic process
mesoderm development
endoderm development
drug catabolic process
response to chemical
exogenous drug catabolic process
response to drug
relationship
is_a
part_of
regulates
qvalue
0.01
0.02
B
ion channel complex
integral component of membrane
transmembrane transporter complex
transporter complex
intrinsic component of membrane
protein complex
membrane protein complex
membrane part
extracellular space
extracellular region
macromolecular complex
extracellular region part
membrane
cell
cellular_component
cell junction
cell part
supramolecular fiber
organelle
organelle part
intracellular
polymeric cytoskeletal fiber
intracellular part
intracellular organelle
intracellular organelle part
non−membrane−bounded organelle
intracellular non−membrane−bounded organelle
cytoskeletal part
intermediate filament
cytoskeleton
intermediate filament cytoskeleton
relationship
is_a
part_of
qvalue
0.01
0.02
0.03
C
inorganic cation transmembrane transporter activity
nucleic acid binding transcription factor activity
cation transmembrane transporter activity
phosphate ion transmembrane transporter activity
cation channel activity
transcription factor activity, sequence−specific DNA binding
inorganic solute uptake transmembrane transporter activity
ion transmembrane transporter activity
RNA polymerase II transcription factor activity, sequence−specific DNA binding
ion channel activity
uptake transmembrane transporter activity
substrate−specific transmembrane transporter activity
substrate−specific channel activity
channel activity
passive transmembrane transporter activity
transmembrane transporter activity
transporter activity
cofactor binding
molecular_function
catalytic activity
binding
heterocyclic compound binding
oxidoreductase activity, acting on paired donors, with incorporation or reduction of molecular oxygen
oxidoreductase activity
organic cyclic compound binding
oxidoreductase activity, acting on paired donors, with incorporation or reduction of molecular oxygen, reduced flavin or flavoprotein as one donor, and incorporation of one atom of oxygen
tetrapyrrole binding
monooxygenase activity
heme binding
steroid hydroxylase activity
qvalue
0.005
0.010
0.015
relationship
is_a

Figure S2. GO Enrichment analysis of the wt vs. mz-*irf6*-/- RNA-seq dataset. Nodes show GO terms associated with enriched genes in the dataset. Edges and arrows show previously annotated relationships between GO terms. Developmental processes such as ectodermal development and otic placode formation are enriched in the analysis, which involve the recruitment of neural crest cell progenitors also involved in palate development. Color represents q-value, with red having a lower q-value and blue having a higher q-value. Q-values were computed using FDR with the Benjamini-Hochberg correction. Data were visualized using the enrichGO and goplot packages in R.

Development • Supplementary information

Figure S3

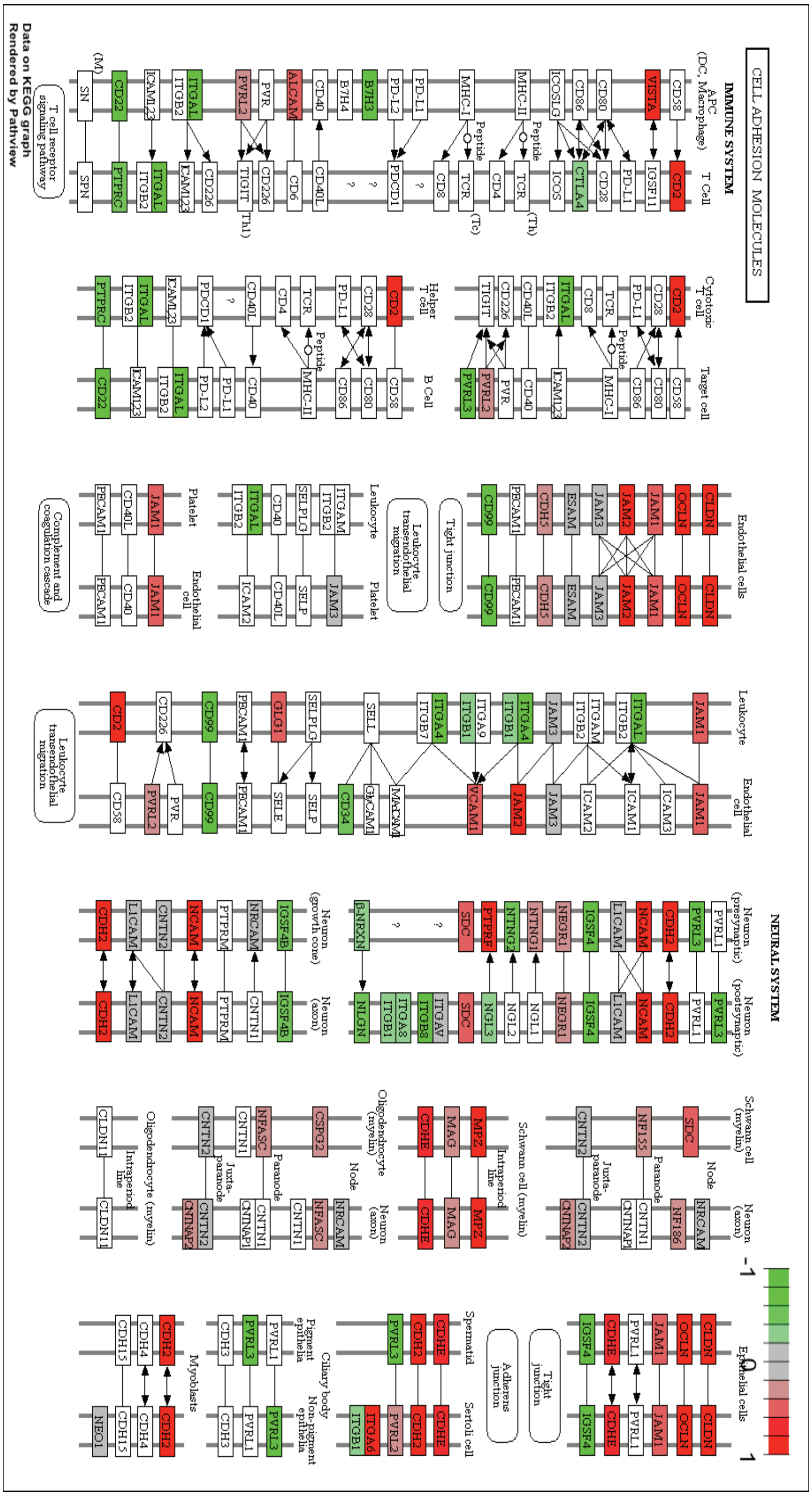
CELL ADHESION MOLECULES
IMMUNE SYSTEM
NEURAL SYSTEM
Data on KEGG graph
Rendered by Pathview

Figure S3. KEGG Graph of Cell Adhesion Molecules. KEGG pathway analysis that maps enriched genes in our dataset to a database containing cellular pathway information. Colored nodes show increased expression of genes in red and decreased expression in green for wild type fish compared to control. Many cell-adhesion molecular interactions are disrupted in the *irf6*-/- embryos. Data were generated using the enrichKEGG and pathview packages in R.

Development • Supplementary information

Figure S4

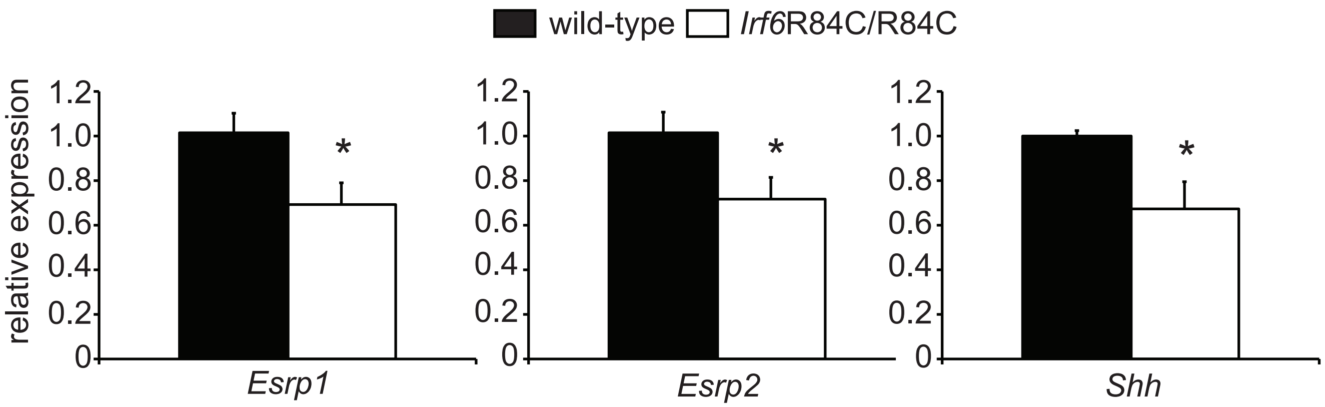

Figure S4. Decreased expression of *Esrp1* and *Esrp2* in *Irf6*R84C mutant mouse embryos. *Irf6*$^{R84C/WT}$ mice were intercrossed, and embryos were collected at E11.5. Embryos were individually genotyped and heads of *Irf6*$^{WT/WT}$ and *Irf6*$^{R54C/R84C}$ embryos were utilized for RNA isolation and RT-qPCR. Expression of *Esrp1*, *Esrp2* and *Shh* were significantly decreased in the *Irf6*$^{R54C/R84C}$ embryos relative to wild type. Expression levels were normalized to *TBP* expression. n=5. Students t-test, $p<0.05$.

Development • Supplementary information

Figure S5

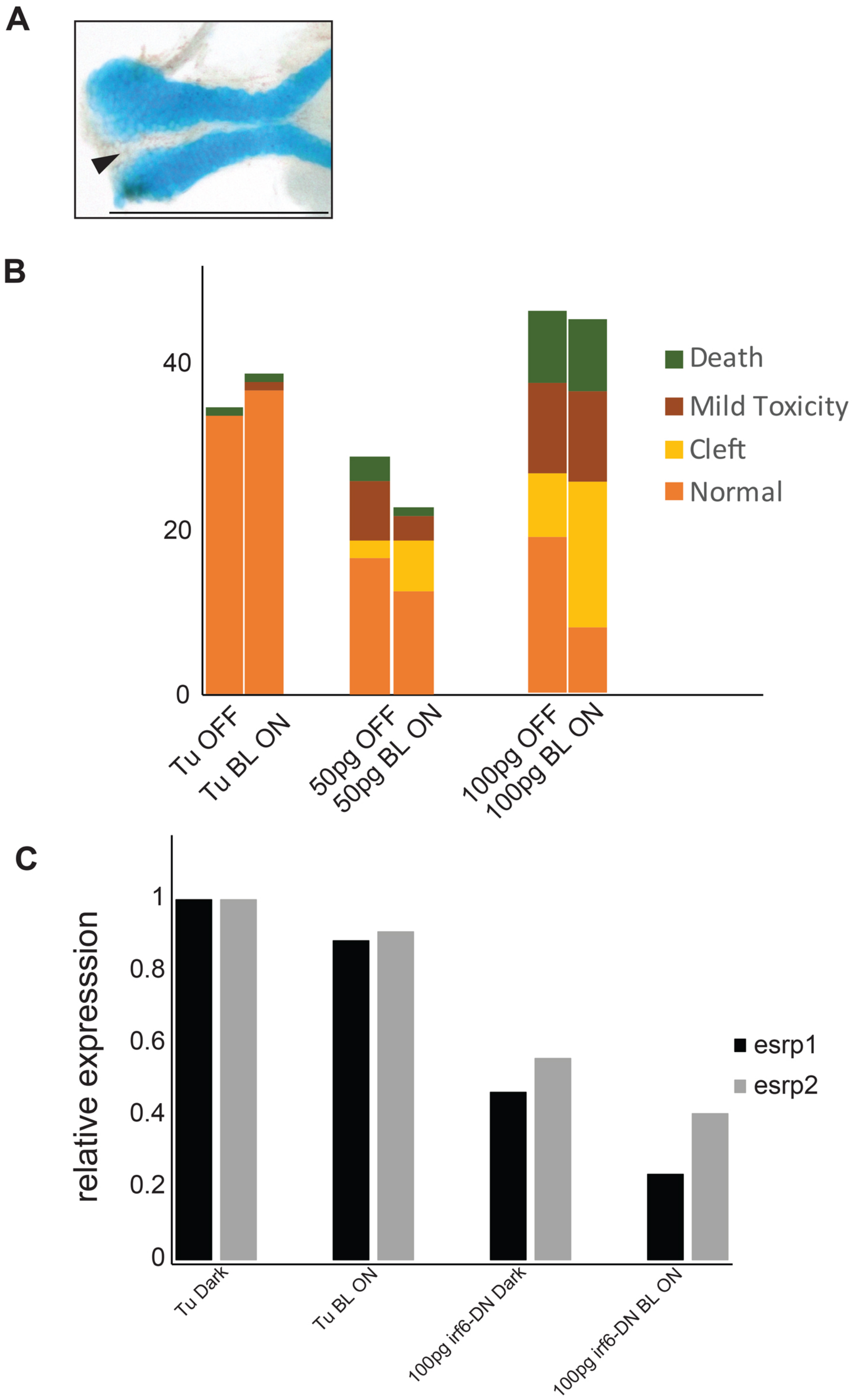
A
B
40
20
0
Death
Mild Toxicity
Cleft
Normal
Tu OFF
Tu BL ON
50pg OFF
50pg BL ON
100pg OFF
100pg BL ON
C
relative expresssion
1
0.8
0.6
0.4
0.2
0
esrp1
esrp2
Tu Dark
Tu BL ON
100pg irf6-DN Dark
100pg irf6-DN BL ON

Figure S5. Optogenetic disruption of *irf6.* (A) Cleft ANC phenotype observed in *irf6*-DN embryos exposed to blue light at 10 hpf. (B) Distribution of observed phenotype in wild type (uninjected) vs. *irf6*-DN injected embryos at 50pg/50pg or 100pg/100pg doses of poly-A tailed EL222-VP16 mRNA and (C120)-*irf6*-DN plasmid grown in the dark or exposed to blue light at 10 hpf. Bar chart height represents absolute number of embryos. (C) qPCR gene expression analysis for *esrp1* and *esrp2*, showing approximately two-fold and five-fold reduction in *esrp1* expression in embryos injected with the optogenetic *irf6-DN* system grown in the dark, or under blue light, respectively. *esrp2* follows a similar trend. Scale bars: 150um.

Development • Supplementary information

Figure S6

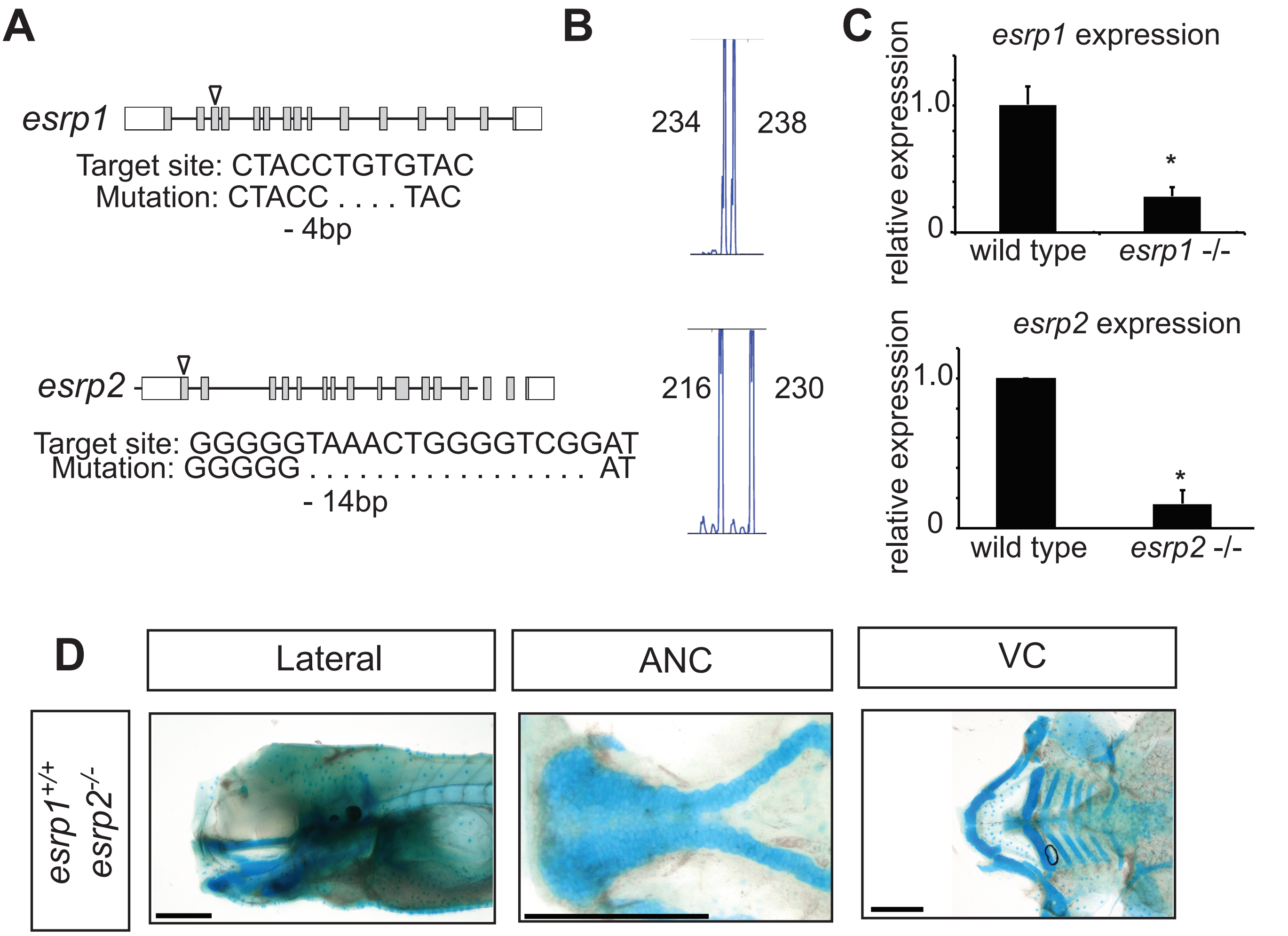

Figure S6. Generation of *esrp1* and *esrp2* CRISPR/Cas9 gene disruption in zebrafish. (A) Schematic representations of *esrp1* and *esrp2* exons, positions of target site (arrow), and sequences of mutations. (B) Micro-satellite results of genotyping PCR showing a size shift consistent with the observed mutation. (C) Expression of the CRISPR/Cas9 targeted gene is decreased relative to wild type, as measured by qPCR. n=3,4. $p<0.01$ (D) *esrp1*$^{+/+}$; *esrp2*$^{-/-}$ fish embryos at 4dpf exhibit normal craniofacial development of the anterior neurocranium and ventral cartilages. Scale bars: 200um.

Figure S8

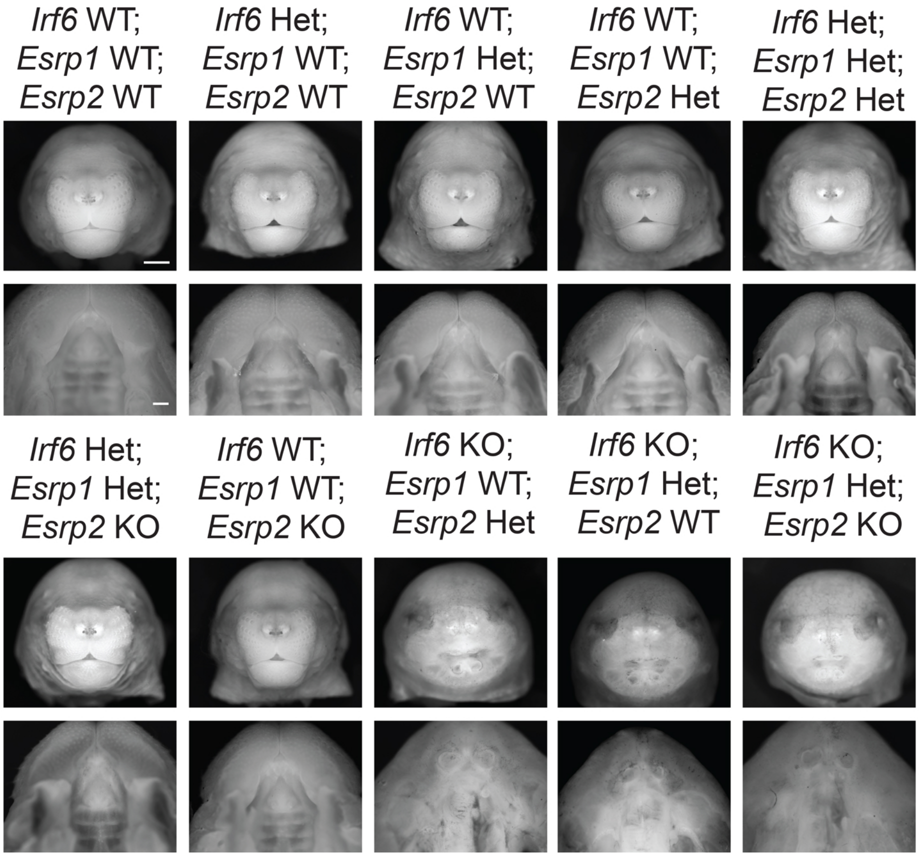

Figure S7. Representative images of *Irf6*$^{R84C/WT}$; *Esrp1*$^{+/-}$; *Esrp2*$^{+/-}$ triple heterozygote in-crosses. Mice compound heterozygous for *Irf6*^{R84C}, *Esrp1* and *Esrp2* were generated by breeding *Irf6*$^{R84C/+}$ with *Esrp1*$^{+/-}$; *Esrp2*$^{-/-}$ mice. The triple heterozygotes were then inter-crossed and embryos were collected at E18.5. Representative frontal and oral images of embryos displaying no craniofacial phenotype in single heterozygotes, triple heterozygotes or *Irf6*$^{R84C/+}$; *Esrp1*$^{+/-}$; *Esrp2*$^{-/-}$. Further, Esrp1/2 alleles did not appear to modify *Irf6*$^{R84C/R84C}$ phenotype, however epithelial adhesions hampered a thorough observation. Scale bar: 100 µm.

Table S1. *Irf6, Esrp1* and *Esrp2* genotypes interact to produce non-Mendelian embryo ratios.

Click here to Download Table S1

Development • Supplementary information

Table S2. Irf6, Esrp1 and Esrp2 genotypes interact to produce non-Mendelian embryo ratios

Irf6	*Esrp1*	*Esrp2*	probability	Expected	Observed
WT	WT	WT	1/64	1.2	3
WT	WT	Het	1/32	2.5	4
WT	WT	KO	1/64	1.2	1
WT	Het	WT	1/32	2.5	2
WT	Het	Het	1/16	4.9	3
WT	Het	KO	1/32	2.5	4
WT	KO	WT	1/64	1.2	0
WT	KO	Het	1/32	2.5	1
WT	KO	KO	1/64	1.2	1
Het	WT	WT	1/32	2.5	5
Het	WT	Het	1/16	4.9	4
Het	WT	KO	1/32	2.5	3
Het	Het	WT	1/16	4.9	10
Het	Het	Het	1/8	9.9	8
Het	Het	KO	1/16	4.9	2
Het	KO	WT	1/32	2.5	2
Het	KO	Het	1/16	4.9	6
Het	KO	KO	1/32	2.5	3
KO	WT	WT	1/64	1.2	1
KO	WT	Het	1/32	2.5	2
KO	WT	KO	1/64	1.2	0
KO	Het	WT	1/32	2.5	5
KO	Het	Het	1/16	4.9	4
KO	Het	KO	1/32	2.5	5
KO	KO	WT	1/64	1.2	0
KO	KO	Het	1/32	2.5	0
KO	KO	KO	1/64	1.2	0

Irf6R84C/WT; *Esrp1+/-*; *Esrp2+/-* triple heterozygous mice were in-crossed and embryos were collected between E12.5 and E21. Table 1 is a subset of expected number of embryos based on Mendelian genetics versus the observed number of viable embryos. The *Irf6R84C/R84C*; *Esrp1-/-* genotype appears to be lethal prior to E12.5 as approximately 5 embryos were expected but zero embryos were observed. A total of 79 embryos were collected from 9 different litters.

Development • Supplementary information
