# Supplementary material for: An Irf6-Esrp1/2 regulatory axis controls midface morphogenesis in vertebrates
Source: Development. 2020 Dec 23;147(24):dev194498. doi: 10.1242/dev.194498 (PMC7774891; doi:10.1242/dev.194498)
Supplement: Reviewer comments [file develop-147-194498_review_history.pdf]

An *Irf6-Esrp1/2* regulatory axis controls midface morphogenesis in vertebrates

Shannon H. Carroll, Claudio Macias-Trevino, Edward B. Li, Kenta Kawasaki, Nikita Myers, Shawn A. Hallett, Nora Alhazmi, Justin Cotney, Russ P. Carstens and Eric C. Liao
DOI: 10.1242/dev.194498

Editor: Patrick Tam

Review timeline
Original submission: 26 June 2020
Editorial decision: 30 July 2020
First revision received: 21 October 2020
Accepted: 2 November 2020

Original submission

First decision letter

MS ID#: DEVELOP/2020/194498

MS TITLE: Requirement of Irf6 and Esrp1/2 in frontonasal and palatal epithelium to regulate craniofacial and palate morphogenesis in mouse and zebrafish

AUTHORS: Shannon H. Carroll, Claudio Macias-Trevino, Edward B-H Li, Kenta C. Kawasaki, Nora Alhazmi, Shawn Hallett, Justin Cotney, Russ P. Carstens, and Eric Chien-Wei Liao

I have now received all the referees' reports on the above manuscript, and have reached a decision. The referees' comments are appended below, or you can access them online: please go to BenchPress and click on the 'Manuscripts with Decisions' queue in the Author Area.

As you will see, the referees express considerable interest in your work, but have some significant criticisms (See also Editor's Note) and recommend a substantial revision of your manuscript before we can consider publication. If you are able to revise the manuscript along the lines suggested, which may involve further experiments, I will be happy receive a revised version of the manuscript (See also Editor's Note). Your revised paper will be re-reviewed by the original referees, and acceptance of your manuscript will depend on your addressing satisfactorily the reviewers' major concerns. Please also note that Development will normally permit only one round of major revision.

We are aware that you may currently be unable to access the lab to undertake experimental revisions. If it would be helpful, we encourage you to contact us to discuss your revision in greater detail. Please send us a point-by-point response indicating where you are able to address concerns raised (either experimentally or by changes to the text) and where you will not be able to do so within the normal timeframe of a revision. We will then provide further guidance. Please also note that we are happy to extend revision timeframes as necessary.

Please attend to all of the reviewers' comments and ensure that you clearly highlight all changes made in the revised manuscript. Please avoid using 'Tracked changes' in Word files as these are lost in PDF conversion. I should be grateful if you would also provide a point-by-point response detailing how you have dealt with the points raised by the reviewers in the 'Response to Reviewers' box. If you do not agree with any of their criticisms or suggestions please explain clearly why this is so.

© 2020. Published by The Company of Biologists under the terms of the Creative Commons Attribution License (https://creativecommons.org/licenses/by/4.0/).

Reviewer 1

Advance summary and potential significance to field

In their manuscript "Requirement of Irf6 and Esrp1/2 in frontonasal and palatal epithelium to regulate craniofacial and palate morphogenesis in mouse and zebrafish" Carroll and colleagues characterize an Irf6-Esrp1/2 genetic pathway in craniofacial development. They used RNA-seq to identify Irf6-regulated genes in zebrafish. They demonstrate that esrp1 is highly dysregulated in embryos lacking irf6 transcripts at 4.5 hpf shortly before these embryos die. They go on to show that Irf6, Esrp1 and Esrp2 are expressed by facial epithelia in both zebrafish and mouse. Zebrafish esrp1;esrp2 double mutants display a midline cleft of the anterior neurocranium that is similar to that observed in dominant-negative Irf6 expressing fish. Using photoactivation they demonstrate that neural crest cells destined for the medial anterior neurocranium migrate appropriately in the double mutant embryos. Rather they find an aberrant population of apparently epithelial cells in the region where the midline of the anterior neurocranium should fuse. They go on to show midfacial defects in the mouse Irf6 mutant and suggest a genetic interaction between Irf6, Esrp1 and Esrp2 in mouse triple mutants based on proposed lethality. All in all, the experiments appear performed well and the results will be of interest to the Development readership.

Comments for the author

There are a few concerns that should be addressed:

Major concerns to be addresseed:

1) The biggest concern relates to the mouse data. Since 18 independent genotypes were examined, it does not seem that obtaining zero Irf6+/-;Esrp1+/-;Esrp2-/- embryos when 3 were expected is very telling about the potential lethality of the genotype. This is particularly true given that more embryos were obtained than expected for the Irf6-/-;Esrp1+/-;Esrp2-/- genotype. As is, the mouse functional data is rather cursory and doesn't strongly support an argument for genetic conservation between zebrafish and mouse. Perhaps a straight-forward test to determine if this genetic pathway is conserved in mouse would be to examine Esrp1 and Esrp2 gene expression in an Irf6 mutant. Alternatively, the zebrafish data do stand on their own and the mouse genetic analyses could be published at another time.

2). Also related to the proposed Irf6-Esrp1/2 genetic pathway, the differential expression is observed in gastrulae stage embryos and only esrp1 is shown. This begs several questions. Is esrp2 regulated by Irf6 at this time? Are esrp2 and irf6 co-expressed in the oral ectoderm (this is only shown for esrp1 and irf6)? Is the expression of esrp1 or esrp2 regulated by Irf6 in the oral ectoderm? This last question could be tested using the Irf6-ENR construct.

3) The authors should show the phenotype of the esrp2 mutant zebrafish.

4) The expression of krt4 in the aberrant cells in the double mutant is not clear in Fig 7.

There are also a few minor comments:

1) There is some confusion regarding the time window of lineage tracing. The authors state that this was performed at the 20-somite stage, similar to Wada, et al. However, Wada labeled cells at 22 hpf, which would be equivalent to 26 somites. 20 somites would be 19 hpf. The authors go on to state that they photoconverted at 12-15 hpf. Would the authors please clarify the timing.

2) In the third paragraph of the introduction, "Epithelial Splicing regulatory factors" should be replaced with "Epithelial Splicing regulatory proteins".

3) In the introduction "…a second CNCC stream that migrates or inferior to the eye…" should be corrected.

© 2020. Published by The Company of Biologists under the terms of the Creative Commons Attribution License (https://creativecommons.org/licenses/by/4.0/).

Reviewer 2

Advance summary and potential significance to field

The paper by Carrol & Trevino et al. presents the analysis of midface and secondary palate development in zebrafish and mice with loss of the Irf6 and Esrp1/2 genes. The results presented are based on the characterization of complementary zebrafish and mouse models with constitutive/global loss of function of the above genes and evaluation of the resulting orofacial phenotypes. The main highlights of the study follow:

1) The Authors found that in zebrafish irf6 controls the expression of esrp1, a gene that has been shown to be required for normal orofacial morphogenesis in the mouse;
2) The Authors describe overlapping expression of Irf6 and Esrp1/2 in the mouse frontonasal prominence ectoderm, lambda periderm, palate and lip epithelium;
3) The Authors show that genetic disruption of irf6 and esrp1/2 in the zebrafish resulted in cleft of the ANC;
4) Based on lineage tracing of the anterior cranial neural crest cells in zebrafish, the Authors purport that cleft of the ANC did not result from migration defects, but from impaired chondrogenesis;
5) Lastly, based on molecular analysis of the aberrant cells localized within the ANC cleft, the Authors report that this cell population expresses both mesenchymal and epithelial markers and is therefore an aberrant mesenchymal/epithelial cell population localized within the cleft.

The study describes a large amount of work that could be of interest to the craniofacial biology community and to the broader developmental biology community interested in processes of tissue fusion.
While orofacial clefts are among the most common human congenital malformations, knowledge of the gene mutations responsible for these maiming birth defects is still rudimentary. Therefore, the present study is warranted and timely.

However, many of the findings reported in this study confirm or extend previously published results. The main strength and novelty of this paper resides in the demonstration that in zebrafish irf6 controls the expression of esrp1 (a gene that has been shown to be required for normal orofacial morphogenesis in the mouse). Thus, this study establishes an irf6-esrp1/2 axis in vertebrate development. Regrettably, the Authors do not highlight this new finding sufficiently in their manuscript. In addition, there is also unease regarding some of the experiments described in the paper, which are not carried out with sufficient rigor.
There are also serious concerns regarding some of the conclusions that are being put forth, which are not supported by adequate experimental evidence and are thus being substantially overstated.

Comments for the author

General Comments:

The study describes a large amount of work that could be of interest to the craniofacial biology community and to the broader developmental biology community interested in processes of tissue fusion. While orofacial clefts are among the most common human congenital malformations, knowledge of the gene mutations responsible for these deforming birth defects is still rudimentary. Therefore, the present study is warranted and timely. However, there is unease regarding some of the findings described in the paper and especially regarding some of the conclusions that are being put forth, which are not supported by adequate experimental evidence and are substantially overplayed.

Specific Comments:

1) The Authors convincingly demonstrate that *irf6* null zebrafish embryos have decreased expression of *esrp1* and that *esrp1* gene expression is dependent on *irf6*, either through direct or indirect regulation in the cephalic epithelium.
In *irf6* null embryos, the RNA-seq results revealed significant downregulation of genes previously known to be downregulated after disruption of *irf6* function (Fig.1B,C). Further, a number of genes associated with human orofacial clefts were also downregulated in *irf6* null embryos (Fig.

© 2020. Published by The Company of Biologists under the terms of the Creative Commons Attribution License (https://creativecommons.org/licenses/by/4.0/).

1B,C). Notably, one of the most downregulated genes in embryos with disruption of *irf6* function was *esrp1*. The identification of the *irf6-esrp* genetic pathway in the developing midface is the most significant findings of the study. This said, the representation of the differentially-expressed genes (DEGs) identified by RNA-seq in wild type relative to mutant (Fig. 1A-C) is somewhat confusing. In the volcano plot, the Authors show that genes like *gata3*, *klf17, ovol1a*, as well as *esrp1*, are expressed at significantly higher level in wild type relative to *irf6-/-*, as stated in the figure legend. This is not consistent with the text (“Results”; page 10), in which the Authors state that these same genes are downregulated in *irf6* mutant embryos *versus* wild type. Consistency between the text, figure, and figure legend is recommended. It is also worthy of note that usually it is best to report downregulation of a specific gene in the mutant *versus* the wild type, rather than describing this finding as upregulation of that gene in the wild type *versus* the mutant. Gene expression in the wild type cannot be considered “upregulated” – as it reflects the normal endogenous expression of that gene in wild type conditions. Therefore the representations of DEGs in the volcano plot should be revised and the figure legend should also be edited to be consistent with the text described in the “Results”; page 10.

2) To confirm the RNA-seq results, the Authors performed qPCR on mz-*irf6*-8bp/-8bp and wild type embryos (“Results”; page 10). Relative to wild type, mz-*irf6*-8bp/-8bp embryos had ~5-fold downregulation of *esrp1* expression. Furthermore, injection of mz-*irf6*-8bp/-8bp embryos with *irf6* mRNA at the 1-cell stage rescued *esrp1* expression, resulting in an increase that was approximately 3-fold higher than wild type (Fig. 1D). Therefore, the Authors established that *esrp1* gene expression is dependent on *irf6*, through direct or indirect regulation. This is an interesting “rescue experiment”; however the Authors do not provide any type of characterization of the rescued phenotype. No details of any kind are given of the rescue experiments.

3) The results obtained by WISH and RNAscope ISH in zebrafish and mouse (shown in Fig 2 and Fig 3) largely confirm previously published findings on co-expression of *Irf6*, *Esrp1* and *Esrp2* in the embryonic oral epithelium surrounding the developing palatal shelves. Also, it has already been reported in the literature that loss of *Irf6* and compound loss of *Esrp1/Esrp2* cause cleft of the secondary palate, and that the lip and primary palate phenotypes differ between *Irf6* and *Esrp1/Esrp2* mutants. The results shown in Fig 2 and Fig 3 are therefore mostly confirmatory, with the exception of the finding that *Irf6* appears to be expressed also in the craniofacial neural crest-derived mesenchyme, which had not been previously reported. This said, it was already known that *Irf6* is expressed also in the neural crest-derived mesenchyme of the pharyngeal arches and in the developing tongue, suggesting a role of *Irf6* in the neural crest beyond its function in the cephalic epithelium.

4) Given the finding that *irf6* appears to be expressed also in the craniofacial neural crest-derived mesenchyme, suggesting a role of *irf6* in the neural crest beyond its function in the cephalic epithelium (see above), a tissue-specific inactivation of *irf6* would be required to unequivocally establish whether the observed orofacial clefting phenotypes result from loss of this transcription factor in the craniofacial neural crest-derived mesenchyme or in the cephalic epithelium. Regrettably, the experiments shown in Fig. 4 do not address this question. The Authors convincingly demonstrate that in their system optogenetic disruption of *irf6* circumvents early embryonic lethality and causes a cleft palate phenotype in zebrafish. However, they fall short of demonstrating whether the function of this transcription factor is essential in the craniofacial mesenchyme or epithelium or both.
- Also, the Authors should state whether this phenotype is fully penetrant, an important information that is not currently present in this manuscript.

5) The results shown in Fig 5 largely confirm previously published findings on the presence of clefting of the lip/primary palate and secondary palate in *Esrp1/Esrp2* compound mutant mice. Zebrafish with compound loss of *esrp1/esrp2*, obtained both by CRISPR/Cas-mediated gene edited and morpholino-mediated gene disruption, exhibit multiple phenotypes including clefting of the anterior neurocranium (ANC).

6) Based on the results shown in Fig. 6A,B, the Authors conclude that *esrp1/2* ablation does not affect the ability of cranial neural crest cells to migrate into the ANC and to reach posterior positions. Therefore, they conclude that clefting of the ANC in *esrp1/2* zebrafish mutants is not

© 2020. Published by The Company of Biologists under the terms of the Creative Commons Attribution License (https://creativecommons.org/licenses/by/4.0/).

due to a lack of progenitor cells, or to abnormal migration of cranial neural crest cells into the ANC. The findings that are currently shown in this figure are interesting, but are not adequate to convincingly support the conclusion that the Authors put forth. Indeed, clefting could result from a decreased number or progenitor cells, or from defects in cell adhesion. Also, migration could still be partially affected in these mutants, thus resulting in a compromised colonization of the ANC by the mutant cranial neural crest cells. The present conclusion is seriously overplayed based on the current experimental findings.

- The Authors should correct the genotype at the top right-hand side of the figure: "*esrp1* MO*; esrp2*" is *esrp2 MO*? Or else?

7) The Authors show a population of cells within the ANC cleft of *esrp1/2* zebrafish mutants (Fig. 7A,B and Fig. 8). These cells are *col2a1* negative, consistent with absent Alcian blue staining. This aberrant cell population expresses *irf6* and *krt4* as well as *Sox10*, therefore exhibiting features of both neural crest-derived mesenchyme and epithelium. This is an interesting finding, demonstrating the presence of a cell population bearing both mesenchymal and epithelial characteristics in the cleft of *esrp1/2* zebrafish mutants. Such a "transitional" cell population has been reported in the literature in mice with orofacial clefting. However, based on the present findings, the Authors conclude that "*epithelial or possibly periderm cells that underly the frontonasal and maxillary prominence derivatives are defective in esrp1/2 null mutants and prevent fusion of the median and lateral elements of the ANC causing a cleft to form*" (("Results"; page 17). This conclusion does not appear to be convincingly supported by the findings shown in Fig. 7A,B and Fig. 8A.

- Of note, the aberrant cell population could also be the result of cell extrusion, as reported in the literature in other systems with abnormal tissue fusion.

- The images shown in Fig. 7A,B have been obtained by using RNAscope ISH, as it is stated in the text and figure legend. As such, the signal should be revealed as a punctate staining, with each punctum corresponding to one molecule of the intended target mRNA, as it is typical of RNAscope ISH. However, either the photographs have been severely overexposed or substantially manipulated, as in most panels puncta are not at all detectable. Better images should be provided.

8) The histological analyses of *Irf6* and *Esrp1/2* knockout mice (Fig. 9) are somewhat superficial and largely confirmatory of previously published results.

9) To further strengthen the presence of an *irf6-esrp1/2* genetic axis, compound mutants *irf6;esrp1;esrp2* with various allelic combinations (including compound triple heterozygosity, as well as heterozygosity for *irf6* and homozygosity for the *esrp* member) were generated. The presence of orofacial clefting (that is absent from single heterozygotes and from single *esrp2* mutants) in some of the allelic combinations produced would unequivocally prove genetic interaction of these genes in midface morphogenesis and palate closure. However, the Authors report lethality prior to E12.5 in some of the allelic combinations they have generated, but do not comment on the potential presence of clefting of the lip and primary palate. Clefting of the lip and primary palate are already detectable in mouse embryos at E11.0. If these mutant embryos survive until E11.0 (regrettably this is not discussed in the present manuscript) it would be important to evaluate their midface morphology. In short, while the Authors can state that there is a general genetic interaction of *Irf6* with *Esrp1* and *Esrp2* in embryonic development, from these experiments, as they are reported, it cannot be unequivocally established whether these genes do interact in murine midface morphogenesis.

10) Absolute statements like: *"the requirement of Irf6 and Esrp1/2 in the frontonasal and palatal epithelium to regulate craniofacial and palate morphogenesis"* (present already in the title of the paper) should be avoided, because they entire study is based on the characterization of constitutive mutants that bear gene loss in all tissues of the embryo. Given the presence of *Irf6* also in craniofacial neural crest-derived mesenchyme, such statements are strongly discouraged because they are not supported by unequivocal experimental evidence.

© 2020. Published by The Company of Biologists under the terms of the Creative Commons Attribution License (https://creativecommons.org/licenses/by/4.0/).

11) **Title**
Among various findings that confirm or extend previously reported results, the main strength of this study resides in the demonstration that in zebrafish irf6 controls the expression of *esrp1*, a gene that has been shown to be required for normal orofacial morphogenesis in the mouse. Regrettably, the current title of the paper does not highlight this interesting finding. The present title (*"Requirement of Irf6 and Esrp1/2 in frontonasal and palatal epithelium to regulate craniofacial and palate morphogenesis in mouse and zebrafish"*) summarizes results that are already known from a large body of published literature. This title fails to highlight the most salient and novel finding of the study. A better title could be: *"An Irf6-Esrp1/2 genetic axis controls midface morphogenesis in vertebrates"*

Minor Comments:
1) Figure 1: In Panel C, it is unclear what the black circles of different diameter under "size" mean. Nodes (represented as colored circles) in the maps appear to have all identical diameters.

2) The alternate use of "anterior neurocranium" and "ANC" throughout the text should be avoided. The Authors should be consistent. If they prefer to use the abbreviation "ANC" then they should use it throughout the text - but should avoid going back to use "anterior neurocranium".

3) Typing errors should be corrected; e.g. "abberant" (page 17) should be changed into "aberrant".

4) A final cartoon or schema summarizing the overall message of the paper should be included within the last figure. This will be very helpful to highlight the overall take-home message of this study.

Reviewer 3

Advance summary and potential significance to field

In this paper "Requirement of Irf6 and Esrp1/2 in frontonasal and palatal epithelium to regulate craniofacial and palate morphogenesis in mouse and zebrafish", Carroll et al. used mouse and zebrafish models to elucidate the role of Irf6-Esrp1/2 signaling in regulating periderm and embryonic epithelium during palate development. The authors provided solid evidence that a RNA-binding protein that regulates alternative splicing in epithelial cells, esrp1, as a novel IRF6 target gene during palate development. They have also further generated transgenic zebrafish model to confirm the function of Esrp1/2 in palate development. These findings are indeed novel and interesting but could benefit from more thorough mechanistic investigations into the observations.

Comments for the author

1) To better reflect the context, the title "Requirement of Irf6 and Esrp1/2 in frontonasal and palatal epithelium to regulate craniofacial and palate morphogenesis in mouse and zebrafish"could be changed to " Irf6-Esrp1/2 signaling in frontonasal and palatal epithelium to regulate craniofacial and palate morphogenesis in mouse and zebrafish".

2) In the second paragraph on page 11, "Further, we detected irf6 and esrp1 transcripts within the same cells, importantly within epithelial cells separating adjacent cartilage elements prior to the fusion of paired Meckel's cartilage elements derived from the mandibular facial prominence (Fig. 2G').". First, this is a mislabeling of 2D' as 2G' since there is no G in Figure 2. Second, without molecular markers, it is difficult to confirm whetherthe cells in arrow pointed region inside the mesenchyme in 2D' are indeed epithelial cells adjacent to cartilage elements. The authors should include epithelial markers to show these cells are epithelial cells. Third, the authors used Sox10 as chondrogenic marker in 2D' but the expression is almost negative in the "adjacent cartilage elements"– could the authors explain this inconsistency?

3) In Figure 2 D-E, it is difficult to see if *irf6* and *esrp1* signals are expressed by the same

© 2020. Published by The Company of Biologists under the terms of the Creative Commons Attribution License (https://creativecommons.org/licenses/by/4.0/).

cells. Could the authors add higher magnification inserts to show that?

4) Also, in page 12, when describing Figure 3, the authors mentioned: "Additionally, with RNAscope, we were able to resolve differences in the mRNA expression pattern of Esrp1 and Esrp2, with Esrp2 generally being more highly expressed in the apical epithelial layer." Again, it is also difficult to see this difference under current magnifications. Could the authors add higher magnification inserts to show that?

5) In page 11, the authors used a "light activated irf6-ENR construct enabled us to control the timing of irf6 disruption by exposing the embryos to a 465nm light-source later in embryogenesis, thereby circumventing gastrulation lethality in the irf6 mutants (Fig. 4A)". In addition to showing the mutants exhibit phenotypes in Figure 4, could the authors also test whether irf6 has been efficiently disrupted in this system?

6) In page 15, there is a typo in the last sentence of the first paragraph "loss of function is lower and requires\d a much smaller dose of MO to generate the cleft ANC phenotype." Please correct "requires\d.

7) In Figure 5C, each genotype has 3 unlabeled images side by side. Could authors add labelling to identify them? Also, in Figure 6C, it will be helpful to add labels in the images to indicate what red and green fluorescent signals represent, respectively.

8) After generating the *esrp1*$^{-4bp/-4bp}$ and *esrp2*$^{-14bp/-14bp}$ mutant zebrafish, did the author also validate efficient loss of esrp1 and esrp2 in addition to the frameshift mutations?

9) Figure 8 is titled "Aberrant anterior neurocranium cells of esrp1/2 double mutants express CNCC and epithelial cell markers". However, in figure 8, the dense population of cells are not stained for any epithelial markers. Although the authors mentioned on page 17 that Fig. 7 showed "this aberrant cell population in the position of the ANC cleft does express irf6 and krt4", however, this co-expression is not visible in the low magnification images in Figure 7B. Could the authors add the missing epithelial cell markers analysis in Figure 8? It is important to confirm whether these cells are epithelial cells or not - this will decide if the phenotypes are cell autonomous or through cell-cell interactions.

10) One very important question remains that how loss of esrp1/2 leads to this ectopic cell mass that is *sox10*+ *col1a1*+*irf6*+? What is the molecular mechanism causing this change?

11) The authors showed the colocalisation of Irf6 and Esrp1/2 in both zebrafish and mouse embryos. They also showed that esrp1/2 are downstream targets of Irf6 in zebrafish. The zebrafish phenotypes of irf6 and esrp1/2 mutants are similar, but mouse phenotypes of Irf6 and Esrp1/2 are different. What is the reason? Are Esrp1/2 downstream of Irf6 in mice? The authors showed the colocalisation in a temporal manner, but didn't show the colocalisation in different regions of the palate at the same stage in mouse. Will the spatial distribution differences explain the phenotype discrepancy in mice?

First revision

Author response to reviewers' comments

Itemized RESPONSEs
MS ID#: DEVELOP/2020/194498
MS TITLE: Requirement of Irf6 and Esrp1/2 in frontonasal and palatal epithelium to regulate craniofacial and palate morphogenesis in mouse and zebrafish

© 2020. Published by The Company of Biologists under the terms of the Creative Commons Attribution License (https://creativecommons.org/licenses/by/4.0/).

Reviewer 1

Reviewer 1 Advance summary and potential significance to field
In their manuscript "Requirement of Irf6 and Esrp1/2 in frontonasal and palatal epithelium to regulate craniofacial and palate morphogenesis in mouse and zebrafish" Carroll and colleagues characterize an Irf6-Esrp1/2 genetic pathway in craniofacial development. They used RNA-seq to identify Irf6-regulated genes in zebrafish. They demonstrate that esrp1 is highly dysregulated in embryos lacking irf6 transcripts at 4.5 hpf, shortly before these embryos die. They go on to show that Irf6, Esrp1 and Esrp2 are expressed by facial epithelia in both zebrafish and mouse. Zebrafish esrp1;esrp2 double mutants display a midline cleft of the anterior neurocranium that is similar to that observed in dominant-negative Irf6 expressing fish. Using photoactivation they demonstrate that neural crest cells destined for the medial anterior neurocranium migrate appropriately in the double mutant embryos. Rather they find an aberrant population of apparently epithelial cells in the region where the midline of the anterior neurocranium should fuse. They go on to show midfacial defects in the mouse Irf6 mutant and suggest a genetic interaction between Irf6, Esrp1 and Esrp2 in mouse triple mutants based on proposed lethality. All in all, the experiments appear performed well and the results will be of interest to the Development readership.

Reviewer 1 Comments for the author

Major concerns to be addressed:

1) The biggest concern relates to the mouse data. Since 18 independent genotypes were examined, it does not seem that obtaining zero Irf6+/-;Esrp1+/-;Esrp2-/- embryos when 3 were expected is very telling about the potential lethality of the genotype. This is particularly true given that more embryos were obtained than expected for the Irf6-/-;Esrp1+/-;Esrp2-/- genotype. As is, the mouse functional data is rather cursory and doesn't strongly support an argument for genetic conservation between zebrafish and mouse. Perhaps a straight-forward test to determine if this genetic pathway is conserved in mouse would be to examine Esrp1 and Esrp2 gene expression in an Irf6 mutant. Alternatively, the zebrafish data do stand on their own and the mouse genetic analyses could be published at another time.

RESPONSE: We have increased the number of mice analyzed and have updated the genotype tables (Table 1, Fig. S7). We discovered that Irf6 heterozygosity modifies the Esrp1 KO and Esrp1/2 dKO phenotype. This data is presented in Fig. 9, which has replaced the previous Fig. 9 of histology. We have also shown decreased expression of Esrp1 and Esrp2 in the Irf6 mutant (Supp Fig. 4), as is the case in the irf6 null zebrafish (Fig.1, Fig. S5). While we do agree with the reviewer that the zebrafish data can stand on its own, we also strongly believe that a paper compare and contrasting both the zebrafish and mouse expression and function of key genes Irf6 and Esrp1/2 tells a more complete story than zebrafish or mouse data on their own.

2). Also related to the proposed Irf6-Esrp1/2 genetic pathway, the differential expression is observed in gastrulae stage embryos and only esrp1 is shown. This begs several questions. Is esrp2 regulated by Irf6 at this time? Are esrp2 and irf6 co-expressed in the oral ectoderm (this is only shown for esrp1 and irf6)? Is the expression of esrp1 or esrp2 regulated by Irf6 in the oral ectoderm? This last question could be tested using the Irf6-ENR construct.

RESPONSE: Fig. 2A-C shows esrp2 expression (by whole mount in situ hybridization) to overlap with the expression of esrp1 and irf6 and to be consistent with the oral epithelium. We did not test esrp2 expression using RNAscope due to the expense of the probe. Further, we have measured esrp1 and 2 expression changes in the Irf6-ENR model of irf6 ablation (Fig S5).

3) The authors should show the phenotype of the esrp2 mutant zebrafish.

RESPONSE: We have added images of the esrp2 mutant zebrafish to Fig. S6). We found no phenotypic changes, similarly to that reported by Burguera et al.

4) The expression of krt4 in the aberrant cells in the double mutant is not clear in Fig 7.

© 2020. Published by The Company of Biologists under the terms of the Creative Commons Attribution License (https://creativecommons.org/licenses/by/4.0/).

RESPONSE: We have updated Fig 7 to include clearer, confocal images. Krt4 is expressed in the periphery of aberrant cells.

There are also a few minor comments:

There is some confusion regarding the time window of lineage tracing. The authors state that this was performed at the 20-somite stage, similar to Wada, et al. However, Wada labeled cells at 22 hpf, which would be equivalent to 26 somites. 20 somites would be 19 hpf. The authors go on to state that they photoconverted at 12-15 hpf. Would the authors please clarify the timing.

RESPONSE: We previously reported sox10:kaede lineage tracing at 19 hpf (20-somites) as using the lineage specific transgenics, we are able to clearly label cranial NCCs at this time point, earlier than Wada et. al. where the lineage tracing was done with di-I injection. We have corrected all discrepancies in timing in the manuscript.

2) In the third paragraph of the introduction, "Epithelial Splicing regulatory factors" should be replaced with
"Epithelial Splicing regulatory proteins".

RESPONSE: This is revised as suggested, thank you. Page 5.

3) In the introduction "…a second CNCC stream that migrates or inferior to the eye…" should be corrected.

RESPONSE: This is revised as suggested, thank you. Page 7.

Reviewer 2

The paper by Carrol & Trevino et al. presents the analysis of midface and secondary palate development in zebrafish and mice with loss of the Irf6 and Esrp1/2 genes. The results presented are based on the characterization of complementary zebrafish and mouse models with constitutive/global loss of function of the above genes and evaluation of the resulting orofacial phenotypes. The main highlights of the study follow:

The Authors found that in zebrafish irf6 controls the expression of esrp1, a gene that has been shown to be required for normal orofacial morphogenesis in the mouse;

The Authors describe overlapping expression of Irf6 and Esrp1/2 in the mouse frontonasal prominence ectoderm, lambda periderm, palate and lip epithelium;

The Authors show that genetic disruption of irf6 and esrp1/2 in the zebrafish resulted in cleft of the ANC;

Based on lineage tracing of the anterior cranial neural crest cells in zebrafish, the Authors purport that cleft of the ANC did not result from migration defects, but from impaired chondrogenesis;

Lastly, based on molecular analysis of the aberrant cells localized within the ANC cleft, the Authors report that this cell population expresses both mesenchymal and epithelial markers and is therefore an aberrant mesenchymal/epithelial cell population localized within the cleft.

General Comments:

The study describes a large amount of work that could be of interest to the craniofacial biology community and to the broader developmental biology community interested in processes of tissue fusion. While orofacial clefts are among the most common human congenital malformations, knowledge of the gene mutations responsible for these deforming birth defects is still rudimentary. Therefore, the present study is warranted and timely. However, there is unease regarding some of the findings described in the paper and especially regarding some of the conclusions that are being

© 2020. Published by The Company of Biologists under the terms of the Creative Commons Attribution License (https://creativecommons.org/licenses/by/4.0/).

put forth, which are not supported by adequate experimental evidence and are substantially overplayed.

Specific Comments:

The Authors convincingly demonstrate that irf6 null zebrafish embryos have decreased expression of esrp1 and that esrp1 gene expression is dependent on irf6, either through direct or indirect regulation in the cephalic epithelium.

In irf6 null embryos, the RNA-seq results revealed significant downregulation of genes previously known to be downregulated after disruption of irf6 function (Fig.1B,C). Further, a number of genes associated with human orofacial clefts were also downregulated in irf6 null embryos (Fig. 1B,C). Notably, one of the most downregulated genes in embryos with disruption of irf6 function was esrp1. The identification of the irf6-esrp genetic pathway in the developing midface is the most significant findings of the study. This said, the representation of the differentially-expressed genes (DEGs) identified by RNA-seq in wild type relative to mutant (Fig. 1A-C) is somewhat confusing. In the volcano plot, the Authors show that genes like gata3, klf17, ovol1a, as well as esrp1, are expressed at significantly higher level in wild type relative to irf6-/-, as stated in the figure legend. This is not consistent with the text (“Results”; page 10), in which the Authors state that these same genes are downregulated in irf6 mutant embryos versus wild type. Consistency between the text, figure, and figure legend is recommended. It is also worthy of note that usually it is best to report downregulation of a specific gene in the mutant versus the wild type, rather than describing this finding as upregulation of that gene in the wild type versus the mutant. Gene expression in the wild type cannot be considered “upregulated” – as it reflects the normal endogenous expression of that gene in wild type conditions. Therefore the representations of DEGs in the volcano plot should be revised and the figure legend should also be edited to be consistent with the text described in the “Results”; page 10.

RESPONSE: This is revised as suggested, thank you. Figure 1.

To confirm the RNA-seq results, the Authors performed qPCR on mz-irf6-8bp/-8bp and wild type embryos (“Results”; page 10). Relative to wild type, mz-irf6-8bp/-8bp embryos had ~5-fold downregulation of esrp1 expression. Furthermore, injection of mz-irf6-8bp/-8bp embryos with irf6 mRNA at the 1-cell stage rescued esrp1 expression, resulting in an increase that was approximately 3-fold higher than wild type (Fig. 1D). Therefore, the Authors established that esrp1 gene expression is dependent on irf6, through direct or indirect regulation. This is an interesting “rescue experiment”; however the Authors do not provide any type of characterization of the rescued phenotype. No details of any kind are given of the rescue experiments.

RESPONSE: Phenotypic analysis of the rescue experiment using irf6 mRNA was previously performed and published (Li et. al. Plos Genetics. Page 11).

The results obtained by WISH and RNAscope ISH in zebrafish and mouse (shown in Fig 2 and Fig 3) largely confirm previously published findings on co-expression of Irf6, Esrp1 and Esrp2 in the embryonic oral epithelium surrounding the developing palatal shelves. Also, it has already been reported in the literature that loss of Irf6 and compound loss of Esrp1/Esrp2 cause cleft of the secondary palate, and that the lip and primary palate phenotypes differ between Irf6 and Esrp1/Esrp2 mutants. The results shown in Fig 2 and Fig 3 are therefore mostly confirmatory, with the exception of the finding that Irf6 appears to be expressed also in the craniofacial neural crest-derived mesenchyme, which had not been previously reported. This said, it was already known that Irf6 is expressed also in the neural crest-derived mesenchyme of the pharyngeal arches and in the developing tongue, suggesting a role of Irf6 in the neural crest beyond its function in the cephalic epithelium.

Given the finding that irf6 appears to be expressed also in the craniofacial neural crest- derived mesenchyme, suggesting a role of irf6 in the neural crest beyond its function in the cephalic epithelium (see above), a tissue-specific inactivation of irf6 would be required to unequivocally establish whether the observed orofacial clefting phenotypes result from loss of this transcription

© 2020. Published by The Company of Biologists under the terms of the Creative Commons Attribution License (https://creativecommons.org/licenses/by/4.0/).

factor in the craniofacial neural crest-derived mesenchyme or in the cephalic epithelium. Regrettably, the experiments shown in Fig. 4 do not address this question. The Authors convincingly demonstrate that in their system optogenetic disruption of irf6 circumvents early embryonic lethality and causes a cleft palate phenotype in zebrafish. However, they fall short of demonstrating whether the function of this transcription factor is essential in the craniofacial mesenchyme or epithelium or both.
Also, the Authors should state whether this phenotype is fully penetrant, an important information that is not currently present in this manuscript.

RESPONSE: All true, we have revised the Discussion to address these points. While the Irf6 floxed allele was reported in the literature, we spent over 2 years trying to obtain it without success. Meanwhile we have generated an independent Irf6 floxed allele and experiments are underway to experimentally test Irf6 function in epithelial (Crect-driven) vs. mesenchymal (Wnt1-Cre-driven). We are also testing at earlier developmental time points than previously reported. This work in progress is beyond the scope of this study, but we do agree with this reviewer that these experiments are needed and that the current data falls short to parse out Irf6 requirement in the mouse between epithelial and non-epithelial lineages.

With regard to optogenetic activation of irf6-ENR, the phenotype is reproducible and robust. The optogenetics experiment is carried out via injection of EL-222 mRNA with the CT120 plasmid driving irf6-ENR, so it is not a germline mutation, where the term “penetrance” is more commonly used to describe the genotype to phenotype correlation. Additionally, as an injection experiment, there can variability of phenotype. However, once optimized, we can reliably observe the cleft phenotype and this quantification is now included (Fig. S5).

Co-expression in the oral epithelium has not been shown and native esrp expression at cellular resolution has not been previously done.

The interaction between Irf6 and Esrp1/2 during development and the earlier lethality of the compound mutant is also novel.

The results shown in Fig 5 largely confirm previously published findings on the presence of clefting of the lip/primary palate and secondary palate in Esrp1/Esrp2 compound mutant mice. Zebrafish with compound loss of esrp1/esrp2, obtained both by CRISPR/Cas-mediated gene edited and morpholino-mediated gene disruption, exhibit multiple phenotypes including clefting of the anterior neurocranium (ANC).

RESPONSE: Lip cleft is novel. This figure sets up the lineage tracing data which is new and mechanistic

Based on the results shown in Fig. 6A,B, the Authors conclude that esrp1/2 ablation does not affect the ability of cranial neural crest cells to migrate into the ANC and to reach posterior positions. Therefore, they conclude that clefting of the ANC in esrp1/2 zebrafish mutants is not due to a lack of progenitor cells, or to abnormal migration of cranial neural crest cells into the ANC. The findings that are currently shown in this figure are interesting, but are not adequate to convincingly support the conclusion that the Authors put forth. Indeed, clefting could result from a decreased number or progenitor cells, or from defects in cell adhesion. Also, migration could still be partially affected in these mutants, thus resulting in a compromised colonization of the ANC by the mutant cranial neural crest cells. The present conclusion is seriously overplayed based on the current experimental findings.

RESPONSE: We have revised our interpretation of this result to cite all these possibilities. Etc.

The Authors should correct the genotype at the top right-hand side of the figure: “esrp1 MO; esrp2” is esrp2 MO? Or else?

RESPONSE: This is revised as suggested, thank you. Figure 6.

The Authors show a population of cells within the ANC cleft of esrp1/2 zebrafish mutants (Fig. 7A,B and Fig. 8). These cells are col2a1 negative, consistent with absent Alcian blue staining. This

© 2020. Published by The Company of Biologists under the terms of the Creative Commons Attribution License (https://creativecommons.org/licenses/by/4.0/).

aberrant cell population expresses irf6 and krt4 as well as Sox10, therefore exhibiting features of both neural crest-derived mesenchyme and epithelium. This is an interesting finding, demonstrating the presence of a cell population bearing both mesenchymal and epithelial characteristics in the cleft of esrp1/2 zebrafish mutants. Such a "transitional" cell population has been reported in the literature in mice with orofacial clefting. However, based on the present findings, the Authors conclude that "epithelial or possibly periderm cells that underly the frontonasal and maxillary prominence derivatives are defective in esrp1/2 null mutants and prevent fusion of the median and lateral elements of the ANC causing a cleft to form" (("Results"; page 17). This conclusion does not appear to be convincingly supported by the findings shown in Fig. 7A,B and Fig. 8A.

RESPONSE: We have revised our interpretation of this result to cite all these possibilities. We have also included additional images to show the expression pattern of these cells, including new sagittal views of the heterotopic cells (Fig. 8B).

Of note, the aberrant cell population could also be the result of cell extrusion, as reported in the literature in other systems with abnormal tissue fusion.
The images shown in Fig. 7A,B have been obtained by using RNAscope ISH, as it is stated in the text and figure legend. As such, the signal should be revealed as a punctate staining, with each punctum corresponding to one molecule of the intended target mRNA, as it is typical of RNAscope ISH. However, either the photographs have been severely overexposed or substantially manipulated, as in most panels puncta are not at all detectable. Better images should be provided.

RESPONSE: The original images in Fig. 7 were obtained using a standard microscope (the confocal core was closed due to the pandemic) and therefore we could not obtain single mRNA resolution. We have reimaged the slides using a confocal microscope and have replaced the images in Fig. 7 with z-stack maximum projections, consistent with the rest of the manuscript.

The histological analyses of Irf6 and Esrp1/2 knockout mice (Fig. 9) are somewhat superficial and largely confirmatory of previously published results.

RESPONSE:
We have removed the histological analyses (Fig. 9) from the manuscript. Instead, we have added analysis of the compound heterozygotes, which tend to have a longer midface, and show that Irf6 heterozygosity modifies the Esrp1 KO and Esrp1/2 dKO (Fig. 9).

To further strengthen the presence of an irf6-esrp1/2 genetic axis, compound mutants irf6;esrp1;esrp2 with various allelic combinations (including compound triple heterozygosity, as well as heterozygosity for irf6 and homozygosity for the esrp member) were generated. The presence of orofacial clefting (that is absent from single heterozygotes and from single esrp2 mutants) in some of the allelic combinations produced would unequivocally prove genetic interaction of these genes in midface morphogenesis and palate closure. However, the Authors report lethality prior to E12.5 in some of the allelic combinations they have generated, but do not comment on the potential presence of clefting of the lip and primary palate. Clefting of the lip and primary palate are already detectable in mouse embryos at E11.0. If these mutant embryos survive until E11.0 (regrettably this is not discussed in the present manuscript) it would be important to evaluate their midface morphology. In short, while the Authors can state that there is a general genetic interaction of Irf6 with Esrp1 and Esrp2 in embryonic development, from these experiments, as they are reported, it cannot be unequivocally established whether these genes do interact in murine midface morphogenesis.

RESPONSE: We have added more mice to this analysis and have found that combinations of Irf6 and Esrp genotypes do not produce orofacial clefts (Fig 9). We predict that Irf6 heterozygocity does not significantly change Esrp expression, as Irf6R84C/R846 mice maintain approximately 70% Esrp expression relative to wild type (Fig. S1).
In addition, we have found that Irf6 heterozygosity can modify the Esrp cleft phenotype, possibly through changes in periderm integrity, as previously published for Irf6 heterozygotes (Richardson et al.).

© 2020. Published by The Company of Biologists under the terms of the Creative Commons Attribution License (https://creativecommons.org/licenses/by/4.0/).

Absolute statements like: “the requirement of Irf6 and Esrp1/2 in the frontonasal and palatal epithelium to regulate craniofacial and palate morphogenesis” (present already in the title of the paper) should be avoided, because they entire study is based on the characterization of constitutive mutants that bear gene loss in all tissues of the embryo. Given the presence of Irf6 also in craniofacial neural crest-derived mesenchyme, such statements are strongly discouraged because they are not supported by unequivocal experimental evidence.

Among various findings that confirm or extend previously reported results, the main strength of this study resides in the demonstration that in zebrafish irf6 controls the expression of esrp1, a gene that has been shown to be required for normal orofacial morphogenesis in the mouse. Regrettably, the current title of the paper does not highlight this interesting finding. The present title (“Requirement of Irf6 and Esrp1/2 in frontonasal and palatal epithelium to regulate craniofacial and palate morphogenesis in mouse and zebrafish”) summarizes results that are already known from a large body of published literature. This title fails to highlight the most salient and novel finding of the study. A better title could be: “An Irf6-Esrp1/2 genetic axis controls midface morphogenesis in vertebrates”

RESPONSE: We agree with the reviewer and have revised the manuscript to eliminate absolute and binary statements pages. We have revised our interpretation of this result to cite all these possibilities. Also we have modified the title.

Minor Comments:
Figure 1: In Panel C, it is unclear what the black circles of different diameter under “size” mean. Nodes (represented as colored circles) in the maps appear to have all identical diameters.

RESPONSE: Diameter size refers to the main nodes, depicted grey. The three presented are similarly sized. We have removed that part of the legend to prevent confusion. (Fig 1).

The alternate use of “anterior neurocranium” and “ANC” throughout the text should be avoided. The Authors should be consistent. If they prefer to use the abbreviation “ANC” then they should use it throughout the text - but should avoid going back to use “anterior neurocranium”.

RESPONSE: Agree and revised on pages 13,14,35,36.

Typing errors should be corrected; e.g. “abberant” (page 17) should be changed into “aberrant”.

RESPONSE: Thank you very much, this is corrected.

A final cartoon or schema summarizing the overall message of the paper should be included within the last figure. This will be very helpful to highlight the overall take-home message of this study.

RESPONSE: Thank you very much, we agree and have rendered a summary figure (Figure 10) we hope you like it.

Reviewer 3

In this paper “Requirement of Irf6 and Esrp1/2 in frontonasal and palatal epithelium to regulate craniofacial and palate morphogenesis in mouse and zebrafish”, Carroll et al. used mouse and zebrafish models to elucidate the role of Irf6-Esrp1/2 signaling in regulating periderm and embryonic epithelium during palate development. The authors provided solid evidence that a RNA-binding protein that regulates alternative splicing in epithelial cells, esrp1, as a novel IRF6 target gene during palate development. They have also further generated transgenic zebrafish model to confirm the function of Esrp1/2 in palate development. These findings are indeed novel and interesting but could benefit from more thorough mechanistic investigations into the observations.

To better reflect the context, the title “Requirement of Irf6 and Esrp1/2 in frontonasal and palatal epithelium to regulate craniofacial and palate morphogenesis in mouse and zebrafish”could be changed to “ Irf6-Esrp1/2 signaling in frontonasal and palatal epithelium to regulate craniofacial and palate morphogenesis in mouse and zebrafish”.

© 2020. Published by The Company of Biologists under the terms of the Creative Commons Attribution License (https://creativecommons.org/licenses/by/4.0/).

RESPONSE: Thank you very much. We have revised the title.

In the second paragraph on page 11, "Further, we detected irf6 and esrp1 transcripts within the same cells, importantly within epithelial cells separating adjacent cartilage elements prior to the fusion of paired Meckel's cartilage elements derived from the mandibular facial prominence (Fig. 2G').". First, this is a mislabeling of 2D' as 2G' since there is no G in Figure 2.

RESPONSE: This has been revised. Pg 11

Second, without molecular markers, it is difficult to confirm whether the cells in arrow pointed region inside the mesenchyme in 2D' are indeed epithelial cells adjacent to cartilage elements. The authors should include epithelial markers to show these cells are epithelial cells. Third, the authors used Sox10 as chondrogenic marker in 2D' but the expression is almost negative in the "adjacent cartilage elements"- could the authors explain this inconsistency?

RESPONSE: We agree and have modified the text to remove comments on this cell population. Regarding the sox10 expression, we have found that RNAscope gives variable expression of sox10. This may be due to fluctuations in sox10 expression in the chondrocytes or possibly due to poor penetration of the RNAscope probe, given the chondrocyte extracellular matrix. However the distinct cellular morphology seen with dapi staining and the well-described craniofacial cartilage structures allows us to be confident in identifying the cartilage elements.

In Figure 2 D-E, it is difficult to see if irf6 and esrp1 signals are expressed by the same cells. Could the authors add higher magnification inserts to show that?

RESPONSE: Whereas we do not have the capability to obtain higher magnifications (these are 63x) we have modified Figure 2 to include larger images of co-expression.

Also, in page 12, when describing Figure 3, the authors mentioned: "Additionally, with RNAscope, we were able to resolve differences in the mRNA expression pattern of Esrp1 and Esrp2, with Esrp2 generally being more highly expressed in the apical epithelial layer." Again, it is also difficult to see this difference under current magnifications. Could the authors add higher magnification inserts to show that?

RESPONSE: We have removed this statement as this trend is difficult to discern in the 2D images.

In page 11, the authors used a "light activated irf6-ENR construct enabled us to control the timing of irf6 disruption by exposing the embryos to a 465nm light-source later in embryogenesis, thereby circumventing gastrulation lethality in the irf6 mutants (Fig. 4A)". In addition to showing the mutants exhibit phenotypes in Figure 4, could the authors also test whether irf6 has been efficiently disrupted in this system?

RESPONSE: We have performed qPCR on zebrafish after optogenetic-mediated irf6 ablation and show changes in esrp1/2 expression (Fig. S5). Further, we previously showed that early induction of the irf6-ENR leads to embryo rupture, which demonstrates that the irf6-ENR construct is effective at inhibiting Irf6 activity (Li et. al. Plos Genetics. Page 11).

In page 15, there is a typo in the last sentence of the first paragraph "loss of function is lower and requires\d a much smaller dose of MO to generate the cleft ANC phenotype." Please correct "requires\d.

RESPONSE: This has been revised. Page 15.

In Figure 5C, each genotype has 3 unlabeled images side by side. Could authors add labelling to identify them? Also, in Figure 6C, it will be helpful to add labels in the images to indicate what red and green fluorescent signals represent, respectively.

RESPONSE: We have revised Figure 5C to clarify that the images are different individuals. We have also revised Figure 6 to include clarifying labels.

© 2020. Published by The Company of Biologists under the terms of the Creative Commons Attribution License (https://creativecommons.org/licenses/by/4.0/).

After generating the esrp1-4bp/-4bp and esrp2-14bp/-14bp mutant zebrafish, did the author also validate efficient loss of esrp1 and esrp2 in addition to the frameshift mutations?

RESPONSE: To validate efficient loss of esrp1 and esrp2 in the CRISPR/Cas9 mutants we confirmed the presence of pre-mature stop codons by sequencing as well as performed qPCR for esrp1 and esrp2 expression. This data is presented in supplemental Fig S5.

Figure 8 is titled “Aberrant anterior neurocranium cells of esrp1/2 double mutants express CNCC and epithelial cell markers”. However, in figure 8, the dense population of cells are not stained for any epithelial markers. Although the authors mentioned on page 17 that Fig. 7 showed “this aberrant cell population in the position of the ANC cleft does express irf6 and krt4”, however, this co-expression is not visible in the low magnification images in Figure 7B. Could the authors add the missing epithelial cell markers analysis in Figure 8? It is important to confirm whether these cells are epithelial cells or not - this will decide if the phenotypes are cell autonomous or through cell-cell interactions.

RESPONSE: We have included additional images of the aberrant cell population co-stained for Irf6, sox10 and krt5 (Fig 9). We are using krt5 as a marker of epithelium. Based on these additional analyses we have modified our interpretation.

One very important question remains that how loss of esrp1/2 leads to this ectopic cell mass that is sox10+ col1a1+irf6+? What is the molecular mechanism causing this change?

RESPONSE: agree this is an important question, we don't have the data for this yet!!!!

The authors showed the co-localization of Irf6 and Esrp1/2 in both zebrafish and mouse embryos. They also showed that esrp1/2 are downstream targets of Irf6 in zebrafish. The zebrafish phenotypes of irf6 and esrp1/2 mutants are similar, but mouse phenotypes of Irf6 and Esrp1/2 are different. What is the reason? Are Esrp1/2 downstream of Irf6 in mice? The authors showed the co-localization in a temporal manner, but didn't show the co-localization in different regions of the palate at the same stage in mouse. Will the spatial distribution differences explain the phenotype discrepancy in mice?

RESPONSE: great questions, we have speculations that are added to the Discussion to compare and contrast mouse and zebrafish cleft models, specifically in the context of Irf6 and Esrp1/2.

The phenotypes of the irf6 and esrp1/2 are dissimilar in that the irf6 mutant embryo ruptures during gastrulation whereas the esrp1/2 mutant is viable up to about 9dpf. It is only possible to observe the zebrafish irf6 cleft phenotype by inducing irf6 knockdown later in development. Generally it is the case in both the zebrafish and the mouse that the Irf6 null phenotype is more severe than the Esrp1/2 null phenotype, consistent with Irf6 being upstream of Esrp. Future experiments where Irf6 is temporally ablated in the mouse could be informative.

Second decision letter

MS ID#: DEVELOP/2020/194498

MS TITLE: An Irf6-Esrp1/2 regulatory axis controls midface morphogenesis in vertebrates

AUTHORS: Shannon H. Carroll, Claudio Macias-Trevino, Edward B. Li, Kenta C. Kawasaki, Nikita Myers, Shawn A. Hallett, Nora Alhazmi, Justin Cotney, Russ P. Carstens, and Eric Chien-Wei Liao
ARTICLE TYPE: Research Article

I am happy to tell you that your manuscript has been accepted for publication in Development, pending our standard ethics checks.

© 2020. Published by The Company of Biologists under the terms of the Creative Commons Attribution License (https://creativecommons.org/licenses/by/4.0/).

Reviewer 1

Advance summary and potential significance to field

The cross species analysis will be of great interest to the Development readership.

Comments for the author

The authors have addressed my previous concerns.

Reviewer 3

Advance summary and potential significance to field

The identification of Irf6-Esrp1/2 pathway and its role in regulating palatogenesis is important. As IRF6 mutation has been frequently associated with facial clefting in humans, this study will provide critical information for future studies.

Comments for the author

All concerns have been adequately addressed.

© 2020. Published by The Company of Biologists under the terms of the Creative Commons Attribution License (https://creativecommons.org/licenses/by/4.0/).
